# Coherence resonance in influencer networks

**DOI:** 10.1038/s41467-020-20441-4

**Published:** 2021-01-04

**Authors:** Ralf Tönjes, Carlos E. Fiore, Tiago Pereira

**Affiliations:** 1 Institute for Physics and Astronomy, University of Potsdam, Karl-Liebknecht-Str. 24, Potsdam, 14476 Germany; 2 Instituto de Física, Universidade de São Paulo, São Paulo, Brazil; 3 Instituto de Ciências Matemáticas e Computação, Universidade de São Paulo, São Carlos, São Paulo, Brazil; 4 Department of Mathematics, Imperial College London, London, SW7 2AZ UK

**Keywords:** Applied mathematics, Nonlinear phenomena

## Abstract

Complex networks are abundant in nature and many share an important structural property: they contain a few nodes that are abnormally highly connected (hubs). Some of these hubs are called influencers because they couple strongly to the network and play fundamental dynamical and structural roles. Strikingly, despite the abundance of networks with influencers, little is known about their response to stochastic forcing. Here, for oscillatory dynamics on influencer networks, we show that subjecting influencers to an optimal intensity of noise can result in enhanced network synchronization. This new network dynamical effect, which we call coherence resonance in influencer networks, emerges from a synergy between network structure and stochasticity and is highly nonlinear, vanishing when the noise is too weak or too strong. Our results reveal that the influencer backbone can sharply increase the dynamical response in complex systems of coupled oscillators.

## Abstract (web summary)

Influencer networks include a small set of highly-connected nodes and can reach synchrony only via strong node interaction. Tönjes et al. show that introducing an optimal amount of noise enhances synchronization of such networks, which may be relevant for neuroscience or opinion dynamics applications.

## Introduction

A central discovery in network science is that a small group of highly connected hubs can couple to the network more strongly than their peers and greatly influence the network behavior^1–6^. Examples of network influencers can be found in neuroscience (e.g., normal and aberrant synaptic connectivity^7–10^), political opinions (e.g., election blogging^11^ or social networks), and man-made scale-free networks (e.g., the internet^1^). Surprisingly, the presence of such influencers makes synchronization of deterministic network dynamics more difficult because networks with influencers require stronger coupling than homogenous networks^12,13^; indeed, in many situations, synchronization of influencer networks cannot be achieved at all^14,15^. This observation is all the more remarkable because synchronization plays a fundamental role in regulating network function^16,17^ and is mediated predominantly through influencers^7,18,19^. This raises a crucial question: why have many real-world networks evolved to contain influencers when they appear to be detrimental to the network dynamics, at least at face value?

While strong random fluctuations usually have a negative effect in complex systems it has long been recognized that a small amount of noise can actually improve the system response and its ability to process information. Known mechanisms for such a constructive influence of noise are stochastic resonance, coherence resonance and noise induced synchronization^20–30^. The term coherence resonance is used to describe an optimal response of noise-induced oscillations without external stimmulus in excitable cells^22^. It was observed in globally coupled systems^23^, in homogeneous networks^24,25^, in non-excitable systems near a Hopf bifurcation^26^ and two coupled oscillators^27^. The effects of coherence resonance and its role in heterogeneous networks such as influencer networks remains elusive.

In this work, we show that stochastic forcing of influencers can lead to an optimal collective network response. Strikingly, introduction of noise synergizes with the network structure to create collective oscillations that become optimal at a given noise strength in the influencers. This phenomenon emerges in two steps. First, the network acts as a nonlinear filter for the stochastic influencer dynamics, and at an optimal noise strength, the influencers induce synchronization in the nodes directly connected to them. Second, different parts of the network develop macroscopic dynamics and interact indirectly through the influencers. We develop an adiabatic theory to uncover this macroscopic interaction law and show that it mediates the emergence of global collective oscillations. When the noise in the influencers is either too weak or too strong, the coupling vanishes. Interestingly, at a macroscopic level, the interaction between different parts of the network can be described by a hyper-graph.

**Fig. 1:**
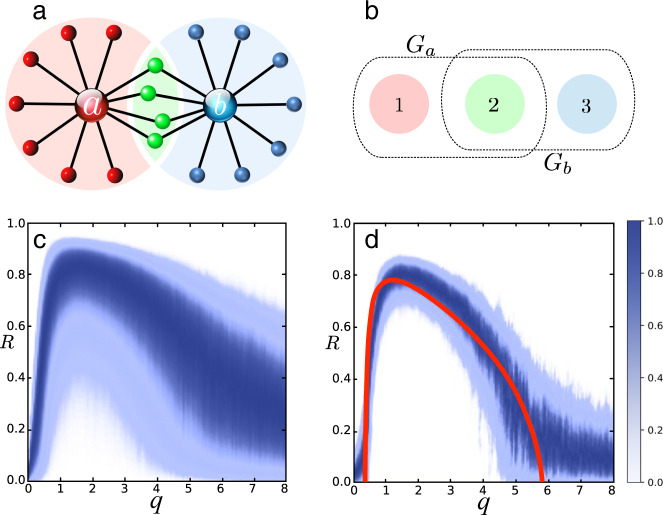
Coherence resonance in an influencer network. Distribution of the order parameter *R* versus the effective diffusion *q* in the influencers. **a** Influencers *a* and *b* are hubs that couple strongly to the network, and all other nodes are regarded as followers. Three distinct partitions of followers are shown in red, blue, and green, which connect to influencer *a*, *b*, and both *a* and *b*, respectively. In our simulation, each partition has 300 followers. **b** Mean-field theory predicts that the interactions of the partition mean-fields take place in a hyper-graph mediated by the coupling functions *G*_*a*_ and *G*_*b*_ see Methods. **c**, **d** For each value of effective noise strength *q* in the influencers, we plot the density of the global order parameter *R* on a color scale from 0 (white) to the maximum value (dark blue). At an optimal noise strength, the mean value of the global order parameter reaches a maximum, revealing the coherence resonance effect. In **c** the dynamical frequency gap between influencers and followers ΔΩ/*λ*_0_ = 18 is moderate, whereas in **d** ΔΩ/*λ*_0_ = 198 is large. The solid red line in **c** is our analytical prediction.

We refer to a network where most nodes couple predominantly to a small number of influencers as an influencer network, and refer to the remaining nodes as followers (Fig. 1). As generic oscillatory dynamics, we consider a network of phase oscillators

1 $${\dot{\vartheta }}_{n}={\omega }_{n}+\frac{{\lambda }_{n}}{{\mu }_{n}}\mathop{\sum }\limits_{m = 1}^{N}{W}_{nm}g({\vartheta }_{m},{\vartheta }_{n})+\sqrt{2{D}_{n}}{\xi }_{n}.$$

Here, *ω*_*n*_ is the natural frequency of node *n*, which couples with strength *λ*_*n*_ to the weighted mean of the coupling functions *g* to neighboring nodes *m*. Given a network weight coupling matrix *W*_*nm*_ ≥ 0, which is nonzero if node *n* receives a link from node *m*, the intensity *μ*_*n*_ is the total coupling weight received by the *n*th node. A table of parameters and their function is provided in Methods. The coupling

2 $$g({\vartheta }_{m},{\vartheta }_{n})=\sin \left({\vartheta }_{m}-{\vartheta }_{n}-\alpha \right)+{c}_{0}$$

is generic for weakly coupled, nearly identical oscillators^31,32^. The parameter *α* is called phase frustration and the bias *c*_0_ is due to shear, an amplitude dependence of the frequency^33,34^. The effect of shear is a shift in the average frequency proportional to the coupling strength. Phase equations with this form of coupling *g* are known as the Kuramoto-Sakaguchi model^35,36^ and are widely applied across scientific disciplines^31–36^. In addition, each term $\sqrt{2{D}_{n}}{\xi }_{n}$ denotes uncorrelated Gaussian white noise of strengths *D*_*n*_. In many studies, the coupling strength *λ*_*n*_ to the local mean-field is chosen to be uniform, in which case the coupling is called normalized. In real-world and experimental systems, though, coupling may be heterogenous and hubs can couple more strongly to the network^5,18^. We model this coupling as

3 $${\lambda }_{n}=\left\{\begin{array}{ll}{\beta }_{n}{\lambda }_{0}&\,\text{for}\; \text{influencers}\,\\ {\lambda }_{0}&\,\text{for}\; \text{followers}\,.\end{array}\right.$$

For simplicity, throughout this exposition we consider the coupling intensity *β*_*n*_ = *β*, the noise strength *D*_*n*_ = *D*, and the frequency *ω*_*n*_ = *ω*, to be identical for all influencers. For the followers we assume a Lorentzian frequency distribution with mean frequency *ω*_0_ and width *γ*_0_. Noise is of identical strength *D*_*n*_ = *D*_0_ in all followers. We denote *Δω* = *ω* − *ω*_0_ the average gap in natural frequencies between influencers and followers.

In Methods we show how Eq. (1) can be recast in terms of dimensionless effective parameters shown in Table 1. These effective parameters, and in particular the influencer effective noise strength *q* = *D*/ΔΩ, play key roles in the collective dynamics of the system. The dynamical frequency gap ΔΩ/*λ*_0_ leads to a time scale separation between the dynamics of the followers and the influencers. A coupling intensity *β* of comparable but smaller magnitude leads to an effective coupling strength *Λ* close to one for which the effect of coherence resonance is most pronounced. We note that the dynamical frequency gap needs to be large in units of *λ*_0_, but it can be small in natural time units. In Supplementary Note 1, we present an example of the transformation for realistic parameters in Eq. (1) to effective parameters.

**Table 1:** Effective dynamical parameters in influencer networks. We obtain these parameters as described in Methods. These are key parameters in the description of coherence resonance and the optimal noise strength in the influencers.

Parameter	Meaning	range
ΔΩ/*λ*_0_ = *Δω*/*λ*_0_ + (*β* − 1)*c*_0_	dynamical frequency gap	ΔΩ/*λ*_0_ ≫ 1
*Λ* = *βλ*_0_/*ΔΩ*	dimensionless coupling strength	*Λ* < 1
*q* = *D*/*ΔΩ*	influencer effective noise strength	*q* = *O*(1)
*D*_0_/*λ*_0_	followers effective noise strength	*D*_0_/*λ*_0_ ≪ 1
*γ*_0_/*λ*_0_	followers frequency heterogeneity	*γ*_0_/*λ*_0_ ≪ 1

We divide the followers into partitions *P*_*σ*_ of nodes connected to the same set of influencers. In Fig. 1, we show an influencer network with two influencers (*a* and *b*) and three partitions of followers (*σ* = 1,2,3), which are connected to influencers *a*, *b*, or both (see additional examples in Supplementary Note 2). To capture the collective dynamics in each partition *σ*, we introduce the complex mean-fields

4 $${Z}_{\sigma }(t)=\frac{1}{| {P}_{\sigma }| }\sum _{n\in {P}_{\sigma }}{e}^{i{\vartheta }_{n}(t)}$$

The modulo of the complex mean-field *R*_*σ*_ = ∣*Z*_*σ*_∣ is the partition order parameter; that is, *R*_*σ*_ = 0 for incoherent, uniformly distributed phases and *R*_*σ*_ = 1 in full synchrony. Similarly, the global mean-field *Z* and order parameter *R* are defined by summing over all followers in the network.

## Results

With deterministic influencers where *q* = 0, and when ∣*Λ*∣ < 1, the influencers cannot frequency lock to the followers. Synchronization of the followers through the influencer backbone is poor and counteracted by noise and frequency heterogeneity in the followers. Our results show that by setting a weak noise strength or frequency heterogeneity in the followers and by changing the effective noise *q* in the influencers, synchronization of the whole network increases, reaches a maximum, and then decreases.

We numerically integrate our model Eq. (1) in dimensionless units (Table 1) for the network with two influencers (as shown in Fig. 1) with 300 identical followers in each partition, and a small fixed noise strength in the followers. By changing the noise strength in the influencers, we then obtain the distribution of the order parameter *R* as a function of *q*. After a transient, the order parameter is independent of the initial conditions. At an optimal noise strength, *R* reaches its maximum (in expected value), as shown in Fig. 1 for ΔΩ/*λ*_0_ = 18 (panel c) and for ΔΩ/*λ*_0_ = 198 (panel d). The solid line is a theoretical prediction in the thermodynamic limit for heterogeneous followers using a slow-fast approximation. Frequency heterogeneity and noise in the followers have qualitatively and quantitatively the same desynchronizing effect. Optimal synchronization of the whole network is predicted theoretically and achieved in all simulations for an effective noise strength *q* ≈ 1 in the influencers, see details in Methods. Our mean-field analysis predicts that the effect of coherence resonance is only observed for very small frequency heterogeneity or noise in the followers, below a threshold that depends on *Λ* (Supplementary Note 3).

**Fig. 2:**
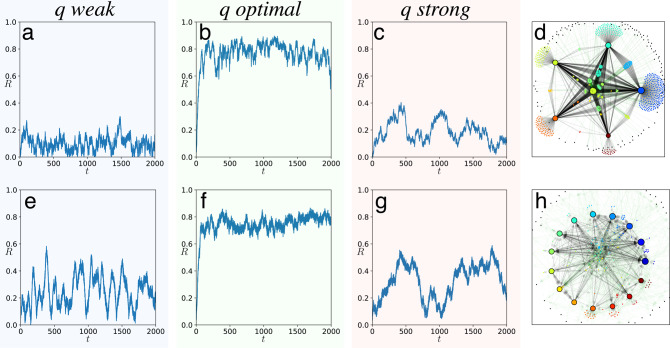
Coherence resonance of the order parameter in different complex networks. **a**–**c**, **e**–**g** The time series of the order parameter *R* for three values of noise strength in the influencers for weak *q* = 0.1 (**a**, **e**), optimal *q* = 1 (**b**, **f**), and strong *q* = 10 (**c**, **g**). **d**, **h** show the corresponding networks with **d** a scale-free network with exponent 2^1^ and **h** C. elegans directed neural network^3^. See Methods for further details. Additional examples can be found in Supplementary Note 5.

In Fig. 2, we show the time series of the order parameter *R* for two complex and real-world networks. The upper row represents a scale-free network and the lower row the directed neural network in the model organism *Caenorhabditis elegans*. We assign the role of influencers to the *K* most strongly connected nodes and use a weighted connectivity matrix *W*_*nm*_ = 1 for all connections from or to an influencer, and *W*_*nm*_ = 0.01 for all other connections. For small effective noise *q* in the influencers (Fig. 2, *q* weak), the order parameter fluctuates at a low level. When *q* = 1 (*q* optimal), the order parameter fluctuates around a value close to 1, revealing coherent collective oscillations. Finally, when *q* is large (*q* strong), the order parameter decreases again, revealing the loss of synchrony. All parameters for the simulation and numerical scheme can be found in Methods. In Supplementary Note 4, we show three additional examples of coherence resonance in influencer networks; with 3 influencers, a random network with 100 influencers, and a network of linked political blogs.

### Stochastic forcing by a single influencer

Let us consider a single influencer. When its followers are asynchronous, the sinusoidal contributions in the sum of the coupling functions for that influencer average out, and the influencer phase is effectively decoupled from the followers. The influencer is independent of the network and acts as a common stochastic force on the followers connected to it. The additive noise in the influencer enters the dynamics of its followers multiplicatively through the coupling function. That is, the network acts as a nonlinear filter for the noise in the influencers. We can show that the effective diffusion constant of the integrated stochastic forcing in the followers, a proxy for the noise strength, attains a maximum at an optimal effective noise strength

5 $${q}_{{\rm{opt}}}=1\ .$$

We present the calculations in Methods and further details in Supplementary Note 5. When oscillations are driven by common multiplicative noise, the effect of noise-induced synchronization can be observed^28–30^. As the common noise intensity is increased the oscillators synchronize faster. This suggests that at this optimal value of *q* the incoherent state will be most unstable. However, behavior of the noise transfer does not explain the synchronization between different partition mean-fields. Because this synchronization requires studying macroscopic dynamics of *R* far from zero, our next goal is to uncover the interaction function between the mean-fields in different partitions.

### Mean-field dynamics of partitions takes place in a hyper-network

For simplicity, we provide an analysis of the influencer network shown in Fig. 1. Mean-field equations for mixed repulsive and attractive coupling or intra- and inter-partition interactions in the followers can be generalized from these results. In Methods, we show that assuming large partition sizes ∣*P*_*σ*_∣ ≫ 1, large dynamical frequency gap *ΔΩ*/*λ*_0_ ≫ 1, and noise free followers *D*_0_ = 0 with frequency heterogeneity *γ*_0_, it is possible to derive averaged dynamics of the partition mean-fields *Z*_*σ*_ in an adiabatic approximation. The resulting deterministic equations have the structure of a hyper-network. In our example, the governing equations are

6 $${\dot{Z}}_{1}=F({Z}_{1},{G}_{a}),\;\; {\dot{Z}}_{2}=F\left(\!{Z}_{2},\frac{1}{2}{G}_{a}+\frac{1}{2}{G}_{b}\!\right)\; \,{\text{and}}\,\; {\dot{Z}}_{3}=F({Z}_{3},{G}_{b})$$

where the coupling functions are

7 $${G}_{a}=G({w}_{a1}{Z}_{1}+{w}_{a2}{Z}_{2})\,\,\,\text{and}\,\,\,{G}_{b}=G({w}_{b2}{Z}_{2}+{w}_{b3}{Z}_{3}).$$

*F* describes a Riccati force (see Methods). The weights *w*_*kσ*_ with ∑_*σ*_*w*_*kσ*_ = 1 denote the relative size of partition *σ* among all followers of influencer *k*. Note that, while in the microscopic description the connections between nodes are pairwise, at the level of mean-fields, edges represented by a coupling function *G*_*k*_ for each influencer can connect multiple partitions of followers. Thus, the mean-field interaction between different parts of the network is described by a hyper-graph.

The interaction functions *G*_*a*_ and *G*_*b*_ can be determined analytically; they depend on *Λ* and *q*, are maximal at an optimal noise strength, and vanish at critical values of *q*. That is, at weak or strong noise in the influencers, the hyper-network interactions vanish, revealing the highly nonlinear nature of the phenomenon. In particular, this means that the macroscopic fields will not interact in the strong noise limit. We derive the analytic expressions for the coupling functions *G*_*k*_(*Z*; *Λ*, *q*) in Methods.

### Global synchronization and resonance

When influencers have equal parameters *q* and *Λ* the synchronization manifold *Z*_*σ*_ = *Z* is invariant under (6) and (7) and stable for phase-attractive coupling. Hence, the macroscopic fields synchronize and we can explain the global coherence resonance by restricting the analysis to this invariant subspace. Our mean-field theory predicts both effects: the coherence resonance of the partition order parameters and phase synchronization of the partition mean-fields as shown in Methods. The solid line in Fig. 1 (right) is the stationary average order parameter predicted by our theory in the infinite time-scale separation limit and with frequency heterogeneity *γ*_0_/*λ*_0_ = 0.02 in the followers. The predicted values agree with simulations of the finite size network, large dynamical frequency gap *ΔΩ*/*λ*_0_ = 198 and identical followers with noise *D*_0_/*λ*_0_ = 0.02. In Supplementary Information, we provide two short movies displaying synchronization of the network in Fig. 1 at an optimal noise strength in the two influencers.

## Discussion

We have found a new effect induced by a synergy between noise and network structure to generate a transition towards a synchronization that would not be possible in the absence of noise. The key element for this effect is the existence of influencers – a group of hubs that couple strongly and connect different parts of a network. Although deterministic network parameters prevent synchronization, we show that an optimal noise strength in the influencers can induce and mediate synchronization. The mechanism for this coherence resonance in influencer networks is different from the known effect of coherence resonance in homogeneous networks with excitatory dynamics, where noise simply excites oscillations^23–25^. At the macroscopic level, the interaction between different parts of the network is indirect and takes place on an emerging hyper-network, thus changing the interaction structure from the microscopic level. Such higher order interactions have previously been conjectured and reported in neuronal data recordings^37^. Our findings suggest that the emergent order in complex systems could be controlled by regulating the noise in only a few key nodes.

## Methods

### Canonical form

To bring the Eq. (1) into a dimensionless form with effective parameters given in Table 1, we change the time scale to units of 1/*λ*_0_ and add the frequency shift from the bias *c*_0_ in the coupling function to the natural frequencies of the oscillators, i.e., *ω*_*n*_ ↦ *ω*_*n*_ + *λ*_*n*_*c*_0_ and $g({\vartheta }_{m},{\vartheta }_{n})\to \sin ({\vartheta }_{m}-{\vartheta }_{n}-\alpha )$. The difference between the average follower frequency and the frequency of an influencer, both including the frequency shift from the coupling bias, is the dynamical frequency gap *ΔΩ*/*λ*_0_ (in units of *λ*_0_). Observing the invariance of the phase equations under a global phase shift, i.e., *ϑ* → *ϑ* − *ω*_0_*t*, we can go into a co-rotating reference frame where the average follower frequency is zero. The deviations of the follower frequencies from their mean frequency *ω*_0_ may be written as *γ*_0_*ν*_*n*_ where the *ν*_*n*_ are taken from some standard distribution with mean zero and the factor *γ*_0_≥0 characterizes the frequency heterogeneity. Then the phase equations for the followers in the new time units and co-rotating reference frame are

8 $${\dot{\vartheta }}_{n}=\frac{{\gamma }_{0}}{{\lambda }_{0}}{\nu }_{n}+\frac{{\lambda }_{n}}{{\lambda }_{0}}\frac{1}{{\mu }_{n}}\sum _{k}{W}_{nk}\sin ({\vartheta }_{k}-{\vartheta }_{n}-\alpha )+\sqrt{2{D}_{n}/{\lambda }_{0}}{\tilde{\xi }}_{n}$$

and for the influencers with phases *ψ*_*k*_

9 $${\dot{\psi }}_{k}=\frac{\Delta {\Omega }_{k}}{{\lambda }_{0}}\left(1+{\Lambda }_{k}\frac{1}{{\mu }_{k}}\sum _{m}{W}_{km}\sin ({\vartheta }_{m}-{\psi }_{k}-\alpha )\right)+\sqrt{2{q}_{k}\frac{\Delta {\Omega }_{k}}{{\lambda }_{0}}}{\tilde{\xi }}_{k}.$$

Here, *Λ*_*k*_ = *λ*_*k*_/*ΔΩ*_*k*_ is the ratio between the coupling strength and the frequency of the influencer. In the noise free case, phase locking is only possible for ∣*Λ*_*k*_∣ > 1. Changing *Λ* can lead to a discontinuous, explosive synchronization^12,38^. The terms ${\tilde{\xi }}_{m}$ are independent white noise with $\langle {\tilde{\xi }}_{m}(t){\tilde{\xi }}_{n}(t^{\prime} )\rangle ={\delta }_{mn}\delta (t-t^{\prime} )$ in the new units of time and *q*_*k*_ is the effective noise strength in the influencers on the fast time scale *ΔΩ*_*k*_/*λ*_0_. In Supplementary Note 1, we provide examples of such rescaling.

### Parameters and their meaning

In Table 2, we present the main parameters that naturally appear in the phase model Eq. (1) and give rise to effective parameters, as shown in Table 1 in the main text. The main parameters in our mean-field analysis are shown in Table 3.

**Table 2:** Parameters in the model presented in Eq. (1) of the main manuscript.

Parameter	Meaning
*ω*_*n*_	isolated frequency of the *n*th oscillator;
	set as *ω*_*n*_ = *ω*_0_ + *γ*_0_*ν*_*n*_ for followers and *ω* for influencers
*ω*_0_	mean frequency of the followers
*γ*_0_*ν*_*n*_	frequency deviation *ω*_*n*_ − *ω*_0_ of the *n*th follower
*γ*_0_	scale parameter of follower frequency distribution
*Δω*	gap (*ω* − *ω*_0_) between influencer and average follower frequency
*W*_*nm*_	nonnegative matrix of connection weights
*μ*_*n*_	connection intensity (*μ*_*n*_ = ∑_*m*_*W*_*nm*_)
*λ*_*n*_	coupling strength of the *n*th oscillator;
	*λ*_0_ for followers and *βλ*_0_ for influencers
*β*	coupling intensity for influencers
*D*_*n*_	noise strength set as *D* for influencers and *D*_0_ for followers
*α*	phase frustration in the coupling function *g*
*c*_0_	shear parameter in the coupling function *g*

**Table 3:** Parameters of the mean-field analysis presented in Eq. (6).

Parameter	Meaning
*P*_*σ*_	follower partitions according to the influencers they connect to
*I*_*σ*_	set of influencers of a partition *σ*
*Z*_*σ*_	complex mean-field of partition *σ* (order parameter *R*_*σ*_ = ∣*Z*_*σ*_∣)
*G*_*k*_	coupling function between mean-fields mediated by influencer *k*
*w*_*kσ*_	relative size of partition *σ* among the followers of influencer *k*
*F*	Ricatti vector field see Eq. (15)
*h*_*σ*_, *h*_*k*_	forces on oscillators in partition *σ* and on influencer *k*
*H*_*σ*_	average of *h*_*σ*_ obtained from adiabatic mean-field approximation

### Simulations and parameter values

In our analysis and our simulations, we use the transformed, dimensionless canonical form (8) and (9) of the phase equations (1). Existing connections in the network from and to the influencers are given the weight *W*_*mn*_ = 1 and connections between followers *W*_*nm*_ = 0.01. The followers couple to their neighbors with strength *λ*_*n*_ = *λ*_0_ and influencers with strength *λ*_*k*_ = *βλ*_0_. The phase frustration in the coupling function is set to *α* = −0.1. The frequency deviations *ν*_*n*_ of the followers are drawn from a Cauchy distribution *p*(*ν*) = 1/*π*(*ν*^2^ + 1) and multiplied by *γ*_0_/*λ*_0_. Thus, frequency heterogeneity and noise strength in the followers are given by *γ*_0_/*λ*_0_ and *D*_0_/*λ*_0_, respectively. We chose *β*_*k*_ = *β*, *λ*_*k*_ = *βλ*_0_ and *D*_*k*_ = *D* for all influencers, so that ΔΩ/*λ*_0_, *Λ* and *q* are identical for all influencers. We integrate the Langevin equations of the phases with an Euler-Maruyama scheme and small time steps *dt* = 5 ⋅ 10^−4^ because of the large time scale separation ΔΩ/*λ*_0_ ≫ 1.

The parameters of Fig. 1 are as follows: The network structure is a pure influencer network without connections between followers or between influencers. We simulate 300 identical followers *γ*_0_/*λ*_0_ = 0 in each of the three partitions with small independent noise *D*_0_/*λ*_0_ = 0.02. In the lower left panel we have *β* = 10, a dynamical frequency gap of ΔΩ/*λ*_0_ = 18 and an effective influencer coupling strength *Λ* = 10/18. In the lower right panel *β* = 100, ΔΩ/*λ*_0_ = 198 and *Λ* = 100/198. For each value of the effective influencer noise strength *q* = *D*/*ΔΩ* we record a histogram of the order parameter over *T* = 10^4^ time units, which is much longer than the relaxation time of *R*. The theoretical prediction, the solid line in the lower right panel, is for noiseless followers *D*_0_ = 0 and *γ*_0_/*λ*_0_ = 0.02.

Parameters of Fig. 2 are as follows: For the *C. elegans* directed neuronal network^3^, we choose the top *K* = 15 out-degree nodes as influencers. All nodes with zero in-degree have been removed, resulting in a network with *N* = 268 nodes. Connections between followers are given the weight *W*_*nm*_ = 0.01. We simulate identical followers *γ*_0_/*λ*_0_ = 0 with small independent noise *D*_0_/*λ*_0_ = 0.02. The dynamical frequency gap between followers and influencers is ΔΩ/*λ*_0_ = 18 and the effective coupling strength in the influencers is *Λ* = 10/18. Shown are three time series of the network order parameter for small (*q* = 0.1), optimal (*q* = 1), and large (*q* = 10) noise strength in the influencers. The undirected scale-free network with exponent 2 is the largest connected component of a network generated via a configurational algorithm^1^ without self loops or double edges. We chose the top 5 degree nodes as influencers. The other parameters are the same as in the *C. elegans* neuronal network.

### Mean-field dynamics in influencer networks

We have developed a mean-field theory for undirected influencer networks with connections exclusively between influencers and followers, as shown in Fig. 1. This theory can be generalized to more complex configurations, heterogenous influencers, directed, attractive, or repulsive coupling between followers and influencers, within partitions or between different partitions. While these generalizations may lead to more complex dynamic behavior, the mechanism for the coherence resonance is apparent in the simplest model.

We consider the network as a union of a set *P* of followers and a set *I* of influencers. The nodes *n* connected to an influencer *k* are elements *n* ∈ *P*_*k*_ of the periphery of the influencer *k*. Intersections of the sets *P*_*k*_ form equivalence classes or partitions *P*_*σ*_ of followers that are connected to the same subsets *I*_*σ*_ of influencers such as in Fig. 1 all followers connected to influencer *a* or *b* or to both influencers. The phases of the oscillators are encoded as complex variables ${z}_{n}=\exp (i{\vartheta }_{n})$ for the followers and ${z}_{k}=\exp (i{\psi }_{k})$ for the influencers. The dynamics can be formulated in terms of partition averages and averages over the influencers of these partitions

10 $${Z}_{\sigma }=\frac{1}{| {P}_{\sigma }| }\sum _{n\in {P}_{\sigma }}{z}_{n}$$

11 $${h}_{\sigma }=\frac{{e}^{-i\alpha }}{2i}\frac{1}{| {I}_{\sigma }| }\sum _{k\in {I}_{\sigma }}{z}_{k}$$

12 $${h}_{k}=\frac{{e}^{-i\alpha }}{2i}\frac{1}{| {P}_{k}| }\sum _{n\in {P}_{k}}{z}_{n}=\frac{{e}^{-i\alpha }}{2i}\sum _{\sigma }{w}_{k\sigma }{Z}_{\sigma }.$$

Here, *h*_*σ*_ and *h*_*k*_ are the forces acting on the followers in partition *σ* and on the influencer *k*. The weight *w*_*kσ*_ is the relative size of partition *σ* in the periphery of an influencer *k*; that is, *w*_*kσ*_ = ∣*P*_*σ*_∣/∣*P*_*k*_∣ when *P*_*σ*_ ⊆ *P*_*k*_ or *w*_*kσ*_ = 0 otherwise. Thus, the phase dynamics (8) and (9) can be written in complex form as

13 $${\dot{z}}_{n}=i{z}_{n}\left({\bar{h}}_{\sigma }{z}_{n}+\frac{{\gamma }_{0}}{{\lambda }_{0}}{\nu }_{n}+{h}_{\sigma }{\bar{z}}_{n}\right)+i{z}_{n}\sqrt{2{D}_{0}/{\lambda }_{0}}{\xi }_{n}(t),\qquad n\in {P}_{\sigma }$$

14 $${\dot{z}}_{k}=i{z}_{k}\frac{\Delta {\Omega }_{k}}{{\lambda }_{0}}\left({\Lambda }_{k}{\bar{h}}_{k}{z}_{k}+1+{\Lambda }_{k}{h}_{k}{\bar{z}}_{k}\right)+i{z}_{k}\sqrt{2{D}_{k}/{\lambda }_{0}}{\xi }_{k}(t)$$

The first reduction of model complexity is via the Ott-Antonsen approach^39^ for followers without Gaussian white noise but frequency heterogeneity *γ*_0_/*λ*_0_ with Cauchy-distributed frequency deviations *ν*_*n*_. In the thermodynamic limit ∣*P*_*σ*_∣ → *∞* (keeping the ratios *w*_*kσ*_ of the partition sizes constant) there exists a globally attractive invariant manifold on which the mean-fields *Z*_*σ*_ evolve by a complex Riccati equation as

15 $${\dot{Z}}_{\sigma }=i\left({\bar{h}}_{\sigma }{Z}_{\sigma }^{2}+i\frac{{\gamma }_{0}}{{\lambda }_{0}}{Z}_{\sigma }+{h}_{\sigma }\right)=F({Z}_{\sigma },{h}_{\sigma }).$$

For large partition sizes, Eqs. (10)–(15) provide a good description of the system dynamics, including an accurate description of the fluctuations of the mean-fields (see Supplementary Note 6). The effect of small noise *D*_0_/*λ*_0_ in the followers is comparable to the effect of frequency heterogeneity *γ*_0_/*λ*_0_. For small white noise, the Ott-Antonsen manifold is no longer invariant but one can derive a hierarchy of corrections to the dynamics (15) in increasing orders of the noise strength. To the zeroth order the effects of frequency heterogeneity and noise are identical^40^. In fact, if the noise in the followers is white Cauchy noise, the equivalence of noise and frequency heterogeneity is exact^41^.

### Slow-fast dynamics

If there is a large dynamical frequency gap ΔΩ_*k*_/*λ*_0_ ≫ 1 between the followers and an influencer, oscillators in the follower group experience an average force from the fast influencer. Conversely, if the followers are desynchronized, the mean-field of the followers vanishes and the influencer phases perform a drift diffusion process on the circle

16 $${\dot{\psi }}_{k}=\frac{\Delta {\Omega }_{k}}{{\lambda }_{0}}+\sqrt{2{D}_{k}/{\lambda }_{0}}{\tilde{\xi }}_{k}(t)$$

whereas the followers connected to only that influencer experience a stochastic forcing by the influencer phase

17 $${\dot{\vartheta }}_{n}=\frac{{\gamma }_{0}}{{\lambda }_{0}}{\nu }_{n}+\sin \left({\psi }_{k}-{\vartheta }_{n}-\alpha \right)+\sqrt{2{D}_{0}/{\lambda }_{0}}{\tilde{\xi }}_{n}(t).$$

This forcing is multiplicative since $\sin \left({\psi }_{k}-{\vartheta }_{n}-\alpha \right)={s}_{k}\cos {\vartheta }_{n}-{c}_{k}\sin {\vartheta }_{n}$ with two uncorrelated but not independent random processes ${s}_{k}(t)=\sin ({\psi }_{k}-\alpha )$ and ${c}_{k}(t)=\cos ({\psi }_{k}-\alpha )$. The diffusion constants *D*_*s*_ and *D*_*c*_ for the integrated stochastic forces quantify an effective noise strength and can be calculated as the integral of the respective autocorrelation functions^42^

18 $$\frac{{D}_{s}}{{\lambda }_{0}}=\frac{{D}_{c}}{{\lambda }_{0}}=\frac{{\lambda }_{0}}{2}\frac{{D}_{k}}{\Delta {\Omega }_{k}^{2}+{D}_{k}^{2}}=\frac{{\lambda }_{0}}{2\Delta {\Omega }_{k}}\frac{{q}_{k}}{1+{q}_{k}^{2}}.$$

We present the details in Supplementary Note 5. By changing the noise strength *D*_*k*_, the effective noise strengths *D*_*s*_ and *D*_*c*_ have a maximum at *D*_*k*_ = ΔΩ_*k*_ or *q*_*k*_ = *D*_*k*_/ΔΩ_*k*_ = 1. At this noise value, and for incoherent followers, the effect of noise-induced synchronization^29^ is expected to be strongest. As the amplitudes of *s*_*k*_ and *c*_*k*_ are bounded, when *D*_*k*_ or the time scale separation *ΔΩ*/*λ*_0_ are further increased the effective noise strengths go to zero.

For *ΔΩ*/*λ*_0_ ≫ 1, the system has slow and fast dynamics and we can replace the influencer phases *z*_*k*_ contributing to the force fields *h*_*σ*_(11) in each partition *σ* by the expected values *G*_*k*_ of *z*_*k*_ subject to Langevin equation (14). On the fast time scale of the influencers, the fields *h*_*k*_ are changing very slowly and can assumed to be constant for the calculation of the *G*_*k*_. In this averaged dynamics, the influencers create an average force *H*_*σ*_ that follows the partition mean-fields adiabatically. The slow dynamics of the partition mean-fields is thus given as

19 $${\dot{Z}}_{\sigma }=i\left({\bar{H}}_{\sigma }{Z}_{\sigma }^{2}+i\frac{{\gamma }_{0}}{{\lambda }_{0}}{Z}_{\sigma }+{H}_{\sigma }\right)=F({Z}_{\sigma },{H}_{\sigma })$$

20 $${H}_{\sigma }=\frac{{e}^{-i\alpha }}{2i}\frac{1}{| {I}_{\sigma }| }\sum _{k\in {I}_{\sigma }}{G}_{k}\left(\sum _{{\sigma }^{\prime}}{w}_{k{\sigma }^{\prime}}{Z}_{{\sigma }^{\prime}};\,{\Lambda }_{k},{q}_{k}\right).$$

This corresponds to a hyper-graph with partitions *σ* as nodes and coupling functions *G*_*k*_ for each edge *k* of the hyper-graph. General setups can be considered as well, with intra and inter-partition coupling and connections between influencers. The absence of such connections shows that the synchronization is indeed a noise-induced effect.

### Mean-field of the fast influencers

The Langevin equation (14) for *z*_*k*_ with constant fields *h*_*k*_ is indeed a complex formulation of the noisy Adler equation^42^

21 $$\dot{\psi }=\frac{\Delta \Omega }{{\lambda }_{0}}\left(1+2\Lambda {\rm{Im}}\left[h{e}^{-i\psi }\right]\right)+\sqrt{2D/{\lambda }_{0}}\xi (t).$$

The expected value *G* of $z=\exp (i\psi )$ is the first circular moment of the stationary distribution which has an expression as a continued fraction^42^ and evaluates to a ratio of confluent hypergeometric limit functions _0_*F*_1_(*o*, *x*)^43^. It can be derived from the Fokker-Planck equation noting that the Fourier modes ${p}_{k}=\langle \exp (ik\psi )\rangle$ of the stationary distribution *p*^*st*^(*ψ*) are in a tridiagonal recurrence relation

22 $$0=ik\Delta \Omega \left(\Lambda \bar{h}{p}_{k+1}+{p}_{k}+\Lambda h{p}_{k-1}\right)-D{k}^{2}{p}_{k}$$

which is solved by a continued fraction. Defining *q* = *D*/*ΔΩ* and

23 $$s=\frac{| h| }{ih},\quad o=1-i\frac{1}{q},\quad {\rm{and}}\quad x=\frac{q}{\Lambda | h| }$$

we have *G* = *p*_1_ and

24 $$G=\frac{1}{o+\frac{{x}^{-2}}{(o+1)+\frac{{x}^{-2}}{(o+2)+\ldots }}}\frac{1}{sx}=\frac{{\,}_{0}{F}_{1}(o+1,{x}^{-2})}{{\,}_{0}{F}_{1}(o,{x}^{-2})}\frac{1}{sox}.$$

### Synchronization manifold and prediction of order parameter

If all influencers have the same effective noise strength $q={q}_{k}=\frac{{D}_{k}}{\Delta {\Omega }_{k}}$ and the same effective coupling strength $\Lambda ={\Lambda }_{k}=\frac{\beta {\lambda }_{0}}{\Delta {\Omega }_{k}}$, the synchronization manifold where all partitions have identical mean-fields *Z*_*σ*_ = *Z* = *R*^*iΘ*^ is invariant under the averaged dynamics (19) and (20) on the hyper-graph and we can write

25 $$\dot{Z}=i\left(\bar{H}{Z}^{2}+i\frac{{\gamma }_{0}}{{\lambda }_{0}}Z+H\right)$$

26 $$H=\frac{{e}^{-i\alpha }}{2i}G\left(Z;\,\Lambda ,q\right)$$

where *G*(*Z*; *Λ*, *q*) is defined as (24) with

27 $$h=\frac{{e}^{-i\alpha }}{2i}Z.$$

In particular, because of rotational symmetry, the dynamics of the amplitude *R* = ∣*Z*∣ does not depend on the angle *Θ* of the mean-field

28 $$\dot{R}={\rm{Re}}\left[\frac{{e}^{-i\alpha }}{2}G(R;\,\Lambda ,q)\right]\left(1-{R}^{2}\right)-\frac{{\gamma }_{0}}{{\lambda }_{0}}R.$$

If the synchronization manifold is stable, the stable fixed points of this dynamics where $\dot{R}=0$ approximate the average order parameter over all followers. From (28) we find that the level sets of the right-hand side of

29 $$\frac{{\gamma }_{0}}{{\lambda }_{0}}={\rm{Re}}\left[\frac{{e}^{-i\alpha }}{2}G(R;\,\Lambda ,q)\right]\frac{1-{R}^{2}}{R}$$

determine this average order parameter *R* for any given *γ*_0_/*λ*_0_ implicitly. We show this prediction for *γ*_0_/*λ*_0_ = 0.02 and *Λ* = 0.51 as a solid line in the lower right panel in Fig. 1. Further resonance curves and maxima of *R* for different heterogeneities *γ*_0_ and different *Λ* can be found in Supplementary Note 3.

## Supplementary information

Supplementary Information

Peer Review File

Description of Additional Supplementary Files

Supplementary Movie 1

Supplementary Movie 2

## Data Availability

The data that support the findings of this study are available from the corresponding author upon reasonable request.

## Code availability

Input files or sets of input parameters for Fortran as well as self-developed Python codes are available from the corresponding author upon request.
