# Supplementary material for: Coherence resonance in influencer networks
Source: Nat Commun. 2021 Jan 4;12:72. doi: 10.1038/s41467-020-20441-4 (PMC7782725; doi:10.1038/s41467-020-20441-4)
Supplement: Supplementary file 1 — Supplementary Information [file 41467_2020_20441_MOESM1_ESM.pdf]

Supplementary Information

Coherence resonance in influencer networks

Ralf Tönjes[1], Carlos Fiore[2] and Tiago Pereira[3,4]

[1] *Institute for Physics and Astronomy, University of Potsdam, Karl-Liebknecht-Str. 24, 14476 Potsdam, Germany*

[2] *Instituto de Física, Universidade de São Paulo, São Paulo, Brazil*

[3] *Instituto de Ciências Matemáticas e Computação, Universidade de São Paulo, São Carlos, São Paulo, Brazil*

[4] *Department of Mathematics, Imperial College London, SW7 2AZ, London, UK*

Supplementary Note 1: Example of transformation into canonical form

We start with weakly non-identical ($\omega_0 = 2.0$, $\Delta\omega/\omega_0 < 10\%$), weakly coupled ($\lambda_0 = 0.01$, $\beta = 10$) phase oscillators with moderately large shear ($c_0 = 0.5$) strong noise ($D = 1.4$) in the hubs and weak noise (or weak frequency heterogeneity) in the followers ($D_0 = 2 \times 10^{-4}$).

$$\dot{\vartheta}_n = 2.0 + \frac{0.01}{\mu_n}\sum_m W_{nm}\left[\sin(\vartheta_m - \vartheta_n - \alpha) + 0.5\right] + \sqrt{4 \times 10^{-4}}\xi_n \tag{1}$$

$$\dot{\vartheta}_{\rm hub} = 2.135 + \frac{0.1}{\mu_{\rm hub}}\sum_m W_{{\rm hub}\ m}\left[\sin(\vartheta_m - \vartheta_{\rm hub} - \alpha) + 0.5\right] + \sqrt{2 \times 1.4}\xi_{\rm hub} \tag{2}$$

After going into a co-rotating reference frame, where the follower mean natural frequency is zero $\vartheta \mapsto \vartheta - (\omega_0 + \lambda_0 c_0)t$ and a change of time scale $t \mapsto \lambda_0 t$, we obtain

$$\dot{\vartheta}_n = \frac{1}{\mu_n}\sum_m W_{nm}\sin(\vartheta_m - \vartheta_n - \alpha) + \sqrt{2 \times 0.02}\tilde{\zeta}_n \tag{3}$$

$$\dot{\vartheta}_{\rm hub} = 18\left[1 + \frac{10}{18}\frac{1}{\mu_{\rm hub}}\sum_m W_{{\rm hub}\ m}\sin(\vartheta_m - \vartheta_{\rm hub} - \alpha)\right] + \sqrt{2 \times \frac{140}{18}18}\tilde{\zeta}_{\rm hub} \tag{4}$$

The effective dimensionless parameters for this system are $\Delta\Omega/\lambda_0 = 18$, $\Lambda = 10/18$ and $q = 140/18 \approx 7$. The large noise strength $D/\lambda_0 = 140$ is only large in units of $\lambda_0 = 0.01$. Here, $D = 1.4$ is already much larger than the optimal noise strength $D_{\rm opt} = 0.18$.

Supplementary Note 2: Hyper-graph

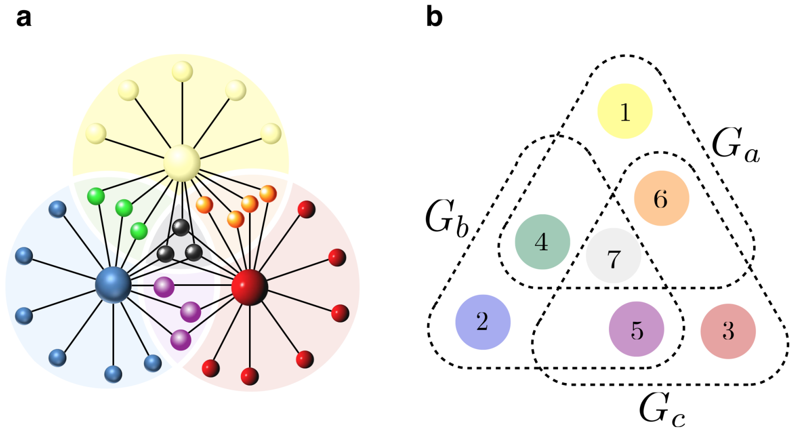

Supplementary Figure 1: **Three-influencer network and hyper-network**. (a) network with three influencers. (b) a hyper-network of seven partitions connected by three hyper-edges.

We discuss the hyper-graph structure emerging at the macroscopic interaction of mean-fields. We will provide illustration of an influencer network with seven partitions of equal size connected through three influencers. After the Ott-Antonsen reduction of the partition mean-field dynamics and after averaging over the fast influencers, the resulting mean-field equations have the following form

$$\dot{Z}_1 = F\left(Z_1, G_a\right), \quad \dot{Z}_2 = F\left(Z_2, G_b\right), \quad \dot{Z}_3 = F\left(Z_3, G_c\right), \tag{5}$$

$$\dot{Z}_4 = F\left(Z_4, \frac{1}{2}G_a + \frac{1}{2}G_b\right), \quad \dot{Z}_5 = F\left(Z_5, \frac{1}{2}G_b + \frac{1}{2}G_c\right), \quad \dot{Z}_6 = F\left(Z_6, \frac{1}{2}G_c + \frac{1}{2}G_a\right), \tag{6}$$

$$\dot{Z}_7 = F\left(Z_7, \frac{1}{3}G_a + \frac{1}{3}G_b + \frac{1}{3}G_c\right) \tag{7}$$

with coupling functions

$$G_a = G\left(\frac{1}{4}Z_1 + \frac{1}{4}Z_4 + \frac{1}{4}Z_6 + \frac{1}{4}Z_7; \Lambda_a, q_a\right) \tag{8}$$

$$G_b = G\left(\frac{1}{4}Z_2 + \frac{1}{4}Z_4 + \frac{1}{4}Z_5 + \frac{1}{4}Z_7; \Lambda_b, q_b\right) \tag{9}$$

$$G_c = G\left(\frac{1}{4}Z_3 + \frac{1}{4}Z_5 + \frac{1}{4}Z_6 + \frac{1}{4}Z_7; \Lambda_c, q_c\right) \tag{10}$$

each connecting four partition mean-fields, and with Riccati dynamics

$$F(Z,G) = \left(\frac{e^{-i\alpha}}{2} G - \frac{\gamma_0}{\lambda_0} Z - \frac{e^{i\alpha}}{2} \bar{G} Z^2 \right) \tag{11}$$

Supplementary Note 3: Impact of follower heterogeneity and noise

In the main body of the manuscript we presented predictions and simulations with fixed values for frequency heterogeneity $\gamma_0/\lambda_0 = 0.02$ or noise strength $D_0/\lambda_0 = 0.02$ in the followers. The influence of these quantities on the coherence resonance can be seen in the mean-field analysis with slow-fast approximation. The frequency heterogeneity can be expressed as a function of all other system parameters, in particular the effective noise strength q and the global order parameter R. The contour plot of γ_0/λ_0 as a function of q and R shows the resonance curves for constant follower heterogeneity (see Supplementary Figure 2).

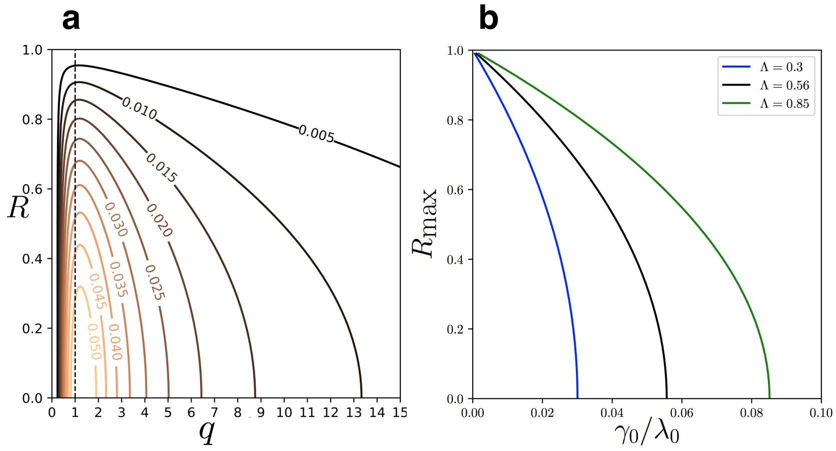

Supplementary Figure 2: **Effect of follower frequency heterogeneity on the coherence resonance**. (a) shows resonance curves of the order parameter as a function of effective noise q in the influencers, with effective coupling $\Lambda = 0.56$. Shown are the level sets of the right-handside. of Eq. (26), which correspond to different frequency heterogeneities γ_0/λ_0 in the followers, as indicated by the values in the labels. Using Gaussian white noise of equal strengths D_0/λ_0 instead of Lorentzian frequency distribution for follower frequencies of width γ_0/λ_0 results in similar resonance curves. (b) shows the maximum value of the order parameter as a function of the frequency heterogeneity for different values of effective coupling strength Λ.

Supplementary Note 4: Examples of coherence resonance

We show three examples of coherence resonance in Supplementary Figure 3. For the three-influencer network motif, we choose each partition with 100 nodes and undirected connections. We show an influencer network of 3000 nodes with 100 influencers. Every node (also the influencers) is connected symmetrically to $k + 1$ randomly selected influencers where k is geometrically distributed with mean $10/6$. Since there are no direct links between followers, all connections have the weight $W_{mn} = 1$. For the directed hyperlink network of 1033 political weblogs after the 2004 US election, we choose the top 8 in-degree nodes as influencers. All nodes with zero out-degree have been removed. Phase coupling in the oscillator dynamics is realized in the opposite direction of the hyperlinks Ref. [11] of the main manuscript. The time scale separation is $\Delta\Omega/\lambda_0 = 18$, $\beta = 10$ ($\Lambda = 10/18$). The noise strength in the followers is $D_0/\lambda_0 = 0.02$ in the three-influencer network and $D_0/\lambda_0 = 0.04$ in the election blog network.

Supplementary Note 5: Effective diffusion

Here, we give an estimate of the effective noise strength for random forces $c(t) = \lambda_0 \cos(\psi - \alpha)$ and $s(t) = \lambda_0 \sin(\psi - \alpha)$ when ψ is a drift-diffusion process

$$\psi(t + \tau) = \psi(t) + \Omega\tau + W_D(\tau) \pmod{2\pi} \tag{12}$$

on the circle with constant velocity Ω, Brownian diffusion $W_D(\tau)$, and diffusion constant D. Such forces act on the follower phases coupled to influencers with phase ψ when the global order parameter is zero, that is, in an incoherent state. The conditional probability density $p(\psi, t + \tau|\psi_0, t)$ is a wrapped normal distribution $p_{WN}(\psi; \mu, \sigma^2)$ with mean $\mu = \psi_0 + \Omega\tau$ and variance $\sigma^2 = 2D\tau$, which in the limit $\tau \to \infty$ becomes a stationary, uniform distribution on the circle. The complex autocorrelation function

$$C(\tau) = \lambda_0^2 \left\langle e^{-i\psi(t)} \cdot e^{i\psi(t+\tau)} \right\rangle_t \tag{13}$$

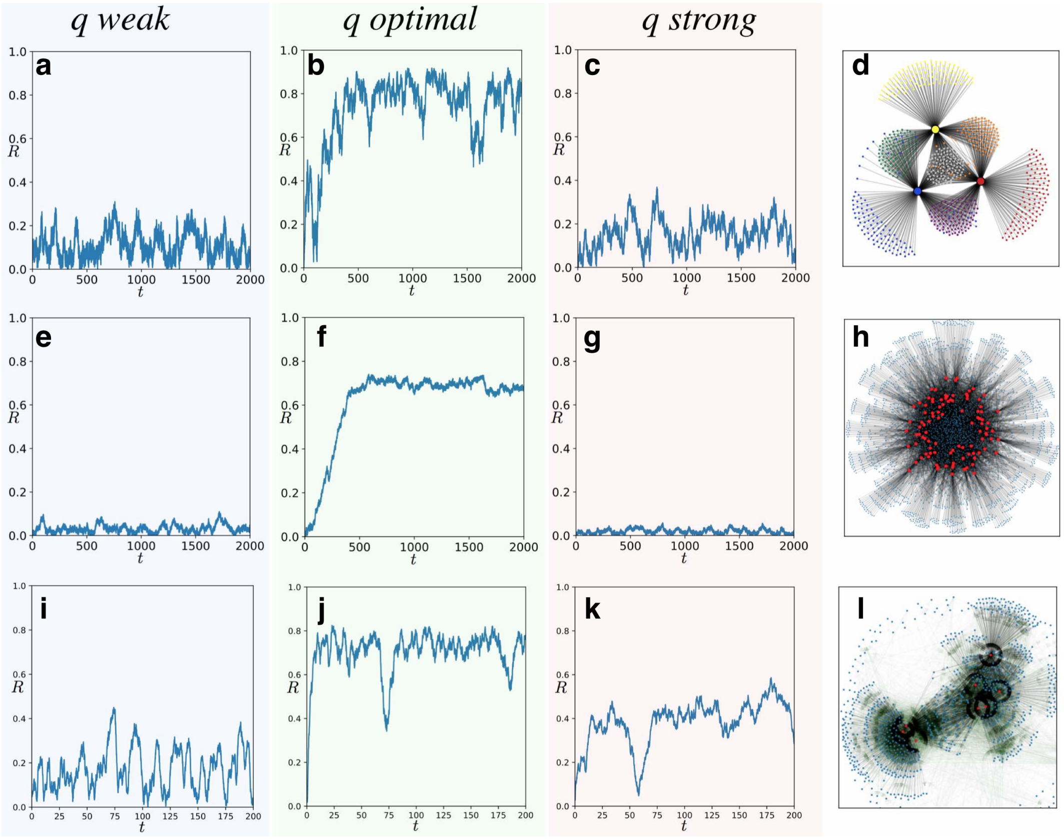

Supplementary Figure 3: **Coherence resonance of the order parameter for different influencer networks**. We show the time series of the order parameter R for three values of noise intensity in the influencers and the network in the corresponding row. Parameters are $\Delta\Omega/\lambda_0 = 18$ and $\Lambda = 10/18$. In (a-d) we show the influencer network with three influencers and seven partitions as shown in SI Note 2, with $D_0/\lambda_0 = 0.02$. In (e-h) we show an influencer network with 3000 nodes including 100 influencers. In (i-l), we show the directed hyperlink network of 1033 political weblogs after the 2004 US election (Ref. [11] of main manuscript) and the top 8-out degree nodes as influencers. Arrows indicate coupling direction which is in the opposite direction of the hyperlinks. The noise strength in the followers is $D_0/\lambda_0 = 0.04$. The noise intensity in the influencers for the simulations in (a,e,i) is weak $q = 0.1$, in (b,f,j) optimal $q = 1.0$, and in (c,g,k) strong $q = 10$.

is the sum of the autocorrelation functions of the two forces $c(t) = \lambda_0 \cos(\psi - \alpha)$ and $s(t) = \lambda_0 \sin(\psi - \alpha)$ in the real part and the cross-correlation function in the imaginary part. Replacing the time average by the average with respect to the conditional probability density and the average over the initial conditions ψ_0 with respect to the stationary probability density, the complex autocorrelation function has the form of the first circular moment of the wrapped

normal distribution

$$C(\tau) = \lambda_0^2 \left\langle e^{i(\Omega\tau + W_D(\tau))} \right\rangle_{p_{WN}} = \lambda_0^2 e^{(i\Omega - D)\tau}. \tag{14}$$

Because of the uniform stationary distribution, the autocorrelation functions of $c(t)$ and $s(t)$ are identical and both forces are uncorrelated; that is $\langle c(t)s(t)\rangle_t = 0$. The time integrals of the random forces are random variables that, according to the central limit theorem, have a variance that grows asymptotically linearly in time. The effective diffusion constant, which is half of the asymptotic speed of this growth, is the integral of the autocorrelation functions

$$D_{\text{eff}} = \frac{\lambda_0^2}{2} \int_0^\infty \text{Re}\left[e^{(i\Omega - D)\tau}\right] d\tau = \frac{\lambda_0^2}{2} \frac{D}{D^2 + \Omega^2} \tag{15}$$

Measuring time in units of $1/\lambda_0$, we obtain the expression in the Methods section of the manuscript.

Supplementary Note 6: Comparison of reduced models

The Ott-Antonsen ansatz for ensembles of phase oscillators with Lorenzian frequency distribution and under common forcing in the zeroth and in the first harmonics results is an exact expression for the dynamics of the mean-field. The forcing can be stochastic if interpreted as Stratonovich stochastic differential equation. In the thermodynamic limit $N \to \infty$ but for finite dynamical frequency gap $\Delta\Omega/\lambda_0$, the fluctuations in the influencer phases lead to fluctuations in the partition mean-fields that are comparable to the partition mean-field fluctuations in the finite size network dynamics. For a large dynamical frequency gap, the effective noise strength of the stochastic forcing vanishes. In the slow-fast approximation, the followers are only subject to the average forces from the influencers, which in turn depend adiabatically on the partition mean-fields. While fluctuations are completely absent in the averaged dynamics, the average forces depend continuously on the influencer effective noise strength $q = D/\Delta\Omega$. Indeed, while for $\Delta\Omega/\lambda_0 = 18$ the averaged dynamics describes the equilibrium order parameters near the optimal noise strength reasonably well, for much larger time scale separation $\Delta\Omega/\lambda_0 = 198$ even the transient to equilibrium is reproduced robustly Supplementary Figure 4.

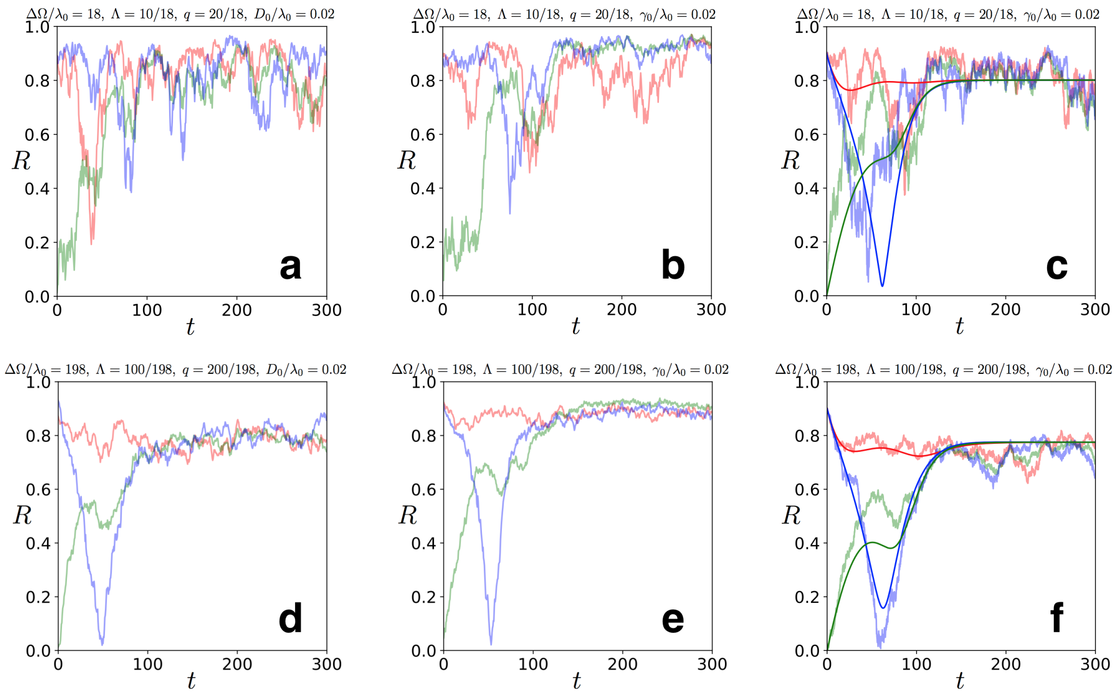

Supplementary Figure 4: **Time series of the order parameters** R_σ (R_1 red, R_2 green, R_3 blue) for the network in Figure 1 of the main manuscript with two influencers for (a-c) moderate time scale separation $\Delta\Omega/\lambda_0 = 18$, noise $q = 20/18$ and reduced coupling strength $\Lambda = 10/18$ and (d-f) large time scale separation $\Delta\Omega/\lambda_0 = 198$ with noise $q = 200/198$ and $\Lambda = 100/198$. Full network simulations with noise $D_0/\lambda_0 = 0.02$ in the followers are shown in (a) and (d). Full network simulations with frequency heterogeneity $\gamma_0/\lambda_0 = 0.02$ in the followers are shown in (b) and (e). In (c) and (f) we show simulations of the Ott-Antonsen mean-field equations under stochastic forcing by the influencers and of the averaged dynamics (smooth curves) obtained under the assumption of infinite time scale separation. The initial conditions are $Z_1 = 0.9\exp(i\pi/2)$, $Z_3 = 0.9\exp(-i\pi/4)$, $Z_3 = 0$, $\psi_a = \pi$ and $\psi_b = -\pi/2$. The drop in the order parameter R_3 is predicted by the averaged dynamics and can be observed in the full network simulations with noise D_0 or frequency heterogeneity γ_0.
