# Supplementary material for: Coherence resonance in influencer networks
Source: Nat Commun. 2021 Jan 4;12:72. doi: 10.1038/s41467-020-20441-4 (PMC7782725; doi:10.1038/s41467-020-20441-4)
Supplement: Supplementary file 3 — Description of Additional Supplementary Files [file 41467_2020_20441_MOESM3_ESM.pdf]

Description of Additional Supplementary Files

Supplementary Movie 1
display the dynamics of noiseless followers with frequency heterogeneity.

Supplementary Movie 2
display the dynamics identical followers with subjected to independent noise.
