# Supplementary material for: Coherence resonance in influencer networks
Source: Nat Commun. 2021 Jan 4;12:72. doi: 10.1038/s41467-020-20441-4 (PMC7782725; doi:10.1038/s41467-020-20441-4)
Supplement: Supplementary file 2 — Peer Review File [file 41467_2020_20441_MOESM2_ESM.pdf]

Reviewer #1 (Remarks to the Author):

The authors introduce a new phenomenon that occurs in so-called influencer networks. Such networks consist of few nodes with many connections and many nodes with comparatively few connections. It is known that such networks are hard to synchronize in the deterministic case. However, as the authors show, the situation changes if noise is introduced into such networks. They show that at an optimal effective noise level, the synchronization level in such networks has a peak before it decreases again, an effect that is associated with coherence resonance. The work is highly relevant for this field, as it raises a number of new questions related to the phenomenon and its possible applications in the context of neuroscience or opinion dynamics. The effects are described and analyzed in a rigorous manner. Apart from numerical simulations, the authors provide a concise mean-field analysis that is able to track the numerical results very nicely. The analysis, interpretations, and conclusions are all well supported and - to the best of my knowledge - contain no flaws or inconsistencies. The methodology is sound and meets all scientific standards. Therefore, I recommend publication of the manuscript after the following comments have been addressed.

1) The term coherence resonance is mainly associated with excitable elements that show the best temporal regularity if exposed to an external noise force. The authors are aware of that, as they mention this in the conclusion. I recommend embedding the discussion of the original term as well as its relationship to the phenomenon discussed here right at the beginning of the paper. Otherwise, the reader might be misled by the title. Here some references to coherence resonance in non-excitable systems would be helpful. For example, the one by Ushakov et al., Phys. Rev. Lett. 95, 123903 (2005). The introduction in my opinion also lacks a discussion on the classical phenomenon of coherence resonance occurring in excitable systems in the context of complex networks. For example, the FitzHugh-Nagumo model studied in the seminal work by A. Pikovky and J. Kurths has been meanwhile investigated for various types of complex networks.

2) The authors introduce a scaling parameter \beta to distinguish the coupling strength of the influencers from the followers. As they introduce new variables to analyze the system, I am not quite sure what the influence of this parameter is. Is it crucial that the followers and the influencers have different coupling strengths?

Anna Zakharova

Reviewer #3 (Remarks to the Author):

The authors analyze a dynamical system of oscillators on a network with influencers (hub nodes) and followers (non-hub nodes). The effect of noise on the influencers is analyzed. The authors show that adding additive noise to the influencers generate a transition towards a synchronization. They call this effect coherence resonance driven by noise. Overall, the idea seems interesting and with potential impact. However, I do have serious concerns about the mathematical analysis done, and I think a major revision is needed for the publication in nature communication.

In general, more motivation and background about the model as well as previews literature is lucking. The authors analyze a version of the Kuramoto model, but the name is not mentioned even once in the main text. In addition, in the definition of the model many parameters are not defined properly.

Questions and remarks about the mean field analysis

What do you assume about the size of partitioning (sets of vertices)? What is the order of limits taken over the sets of vertices P_a and P_b, do they all go together to infinity? do you maintain the ratio between them fixed?
How does the number of the intersecting nodes affect the coherence resonance?

In the mean field analysis, it is not clear to me what is the assumption on the size of the set of nodes that interact with both hubs, do you assume that this set goes to infinity as well? In what rate with respect to the other sets P_a, and P_b? Or do you assume that there is only one such node? Otherwise, it is not clear to me how Eq. (4) is derived. The same comments are valid for Eq. (17).
The author claim that the point of optimal q is an unstable point. Is this shown analytically a stability analysis of the model? How will that change with the number of influencer and followers?
In sec 2.3.2. Slow fast dynamics, Eq. (16) is valid for $D_0=0$ and large partitioning, these assumptions should be stated in the methods section as well.
In line 157, Under what assumption the field h_σ is constant? When will that be valid? Constant with respect to what?
What is the assumption on the noise strength for the followers, D_0? Is there a threshold for which we see the coherence resonance? Does the effect breakdown for high noise? What is the optimal q for D_0 non-zero when there is no frequency heterogeneity?
From the analysis, the effect of coherence resonance driven by noise can be seen from the analysis of a single influencer. What is the effect of having more influencers? And the interaction between them through the followers in both groups? Is there an assumption on the number of influencers for which this effect is apparent? Will it be enough to add noise only to one of the influencers to see this effect?
How does these results depend on the initial condition of the model?

Questions and remarks about the simulations:

Figure 1:
How the coherence resonance driven by noise effect depends on the number of influencers and followers? A graph showing how the optimal q changes as a function of the number of influencers can be nice.
What is the difference in the parameters between the two figures?
How many nodes are connected to both influencers a and b?
Is there a threshold for D_0 for which you start see the synchronization effect?
How do you determine the time T ?

Figure 2:
Which nodes are considered as influencers and which one are the followers how do you determine the value of the noise for each node?
How does R(q) looks like for these networks?
What did we learn from these examples? Why should I expect that the optimal q is the same for these different configurations? Is this value universal?

Minor comments:

Definition of the model – some parameters in the model are not well-defined.
The matrix W_{nm} can the weight be negative what are the range of values for possible weights?
It is not clear whether you consider uniform diffusion coefficient, D_i or does it change between nodes? In Eq. (1) the value is different for each node, but in the paragraph after it, a constant D is considered without stating it.
The same for the noise strength, q, also it is not defined properly in the main text.
$\Delta\omega$ Is not defined. Should that be γ_0?
What is the frequency distribution of the influencers with respect to followers?
The shifts in the frequencies is not clear, it is not clear what is assumed about the initial distribution of frequencies for the follower and influencers. A better explanation in the supplementary material stating all the distribution and the transformation properly is needed.
The manuscript contains many typos. The authors must check the manuscript carefully.

Reviewer #1 (Remarks to the Author):

The authors introduce a new phenomenon that occurs in so-called influencer networks. Such networks consist of few nodes with many connections and many nodes with comparatively few connections. It is known that such networks are hard to synchronize in the deterministic case. However, as the authors show, the situation changes if noise is introduced into such networks. They show that at an optimal effective noise level, the synchronization level in such networks has a peak before it decreases again, an effect that is associated with coherence resonance. The work is highly relevant for this field, as it raises a number of new questions related to the phenomenon and its possible applications in the context of neuroscience or opinion dynamics. The effects are described and analyzed in a rigorous manner. Apart from numerical simulations, the authors provide a concise mean-field analysis that is able to track the numerical results very nicely. The analysis, interpretations, and conclusions are all well supported and - to the best of my knowledge - contain no flaws or inconsistencies. The methodology is sound and meets all scientific standards. Therefore, I recommend publication of the manuscript after the following comments have been addressed.

We thank the reviewer for the time spent evaluating the work and careful read of the manuscript which helps us to improve manuscript.

1) The term coherence resonance is mainly associated with excitable elements that show the best temporal regularity if exposed to an external noise force. The authors are aware of that, as they mention this in the conclusion. I recommend embedding the discussion of the original term as well as its relationship to the phenomenon discussed here right at the beginning of the paper. Otherwise, the reader might be misled by the title. Here some references to coherence resonance in non-excitable systems would be helpful. For example, the one by Ushakov et al., Phys. Rev. Lett. 95, 123903 (2005). The introduction in my opinion also lacks a discussion on the classical phenomenon of coherence resonance occurring in excitable systems in the context of complex networks. For example, the FitzHugh-Nagumo model studied in the seminal work by A. Pikovky and J. Kurths has been meanwhile investigated for various types of complex networks.

We now emphasize oscillator networks in the abstract and added a paragraph in the introduction (line 47-52) including relevant citations.

2) The authors introduce a scaling parameter \beta to distinguish the coupling strength of the influencers from the followers. As they introduce new variables to analyze the system, I am not quite sure what the influence of this parameter is. Is it crucial that the followers and the influencers have different coupling strengths?

The parameter \beta is important in the time scale separation. \Lambda is the ratio of \beta and the dynamical frequency gap and should be smaller than one, but not much smaller (lines 83-89).

Reviewer #3 (Remarks to the Author):

The authors analyze a dynamical system of oscillators on a network with influencers (hub nodes) and followers (non-hub nodes). The effect of noise on the influencers is analyzed. The authors show that adding additive noise to the influencers generates a transition towards a synchronization. They call this effect coherence resonance driven by noise. Overall, the idea seems interesting and with potential impact. However, I do have serious concerns about the mathematical analysis done, and I think a major revision is needed for the publication in nature communication.

We thank the reviewer for the thoughtful comments and efforts to improve the manuscript. We have included explanations about the model as requested throughout the main text as well as a table of parameters in the Methods. More examples are given in the Supplemental Materials.

In general, more motivation and background about the model as well as previous literature is lacking.

We have added a background on coherence resonance (lines 47-52) as well as citations to the Kuramoto-Sakaguchi model and its importance (lines 67-69).

The authors analyze a version of the Kuramoto model, but the name is not mentioned even once in the main text.

We now explicitly mention it in the text along with citations (line 67-69).

In addition, in the definition of the model many parameters are not defined properly.

We provide an exposition of the parameters (lines 75-80) and discuss the physical meaning of the effective parameters (lines 83-89). We also added tables 2 and 3 of model parameters and their meaning in the Methods.

Questions and remarks about the mean field analysis
- What do you assume about the size of partitioning (sets of vertices)?
- What is the order of limits taken over the sets of vertices P_a and P_b, do they all go together to infinity? Do you maintain the ratio between them fixed?
- How does the number of the intersecting nodes affect the coherence resonance?
In the mean field analysis, it is not clear to me what is the assumption on the size of the set of nodes that interact with both hubs, do you assume that this set goes to infinity as well?
- In what rate with respect to the other sets P_a, and P_b?

The questions concern the so-called thermodynamic limit of infinite network size. The partitions (sets of followers) need to be moderately large ~100 for the Ott-Antonsen approach to be a good approximation. The reviewer is correct. We assume in the mean-field analysis the ratios between the partition sizes to be fixed as |P_1|, |P_2| and |P_3| go to infinity (line 298). The relative size of a partition in the set of followers of an influencer determines the contribution of that partition to the coupling function in the hypergraph (line 291).

Or do you assume that there is only one such node? Otherwise, it is not clear to me how Eq. (4) is derived. The same comments are valid for Eq. (17).

The partition P_2 of intersection nodes must have a macroscopic size, otherwise Z_1 and Z_3 are decoupled in the thermodynamic limit.

The authors claim that the point of optimal q is an unstable point. Is this shown analytically a stability analysis of the model?

We do not perform a stability analysis of the ODEs in the adiabatic approximation but observe stability in numerical integration (Suppl. Mat. Sec. D and F). The stability analysis for Eq. (25) within the synchronization manifold is straight forward. We expect it may be possible to get the (transversal) stability of the synchronization manifold from the general properties of the hypergraph Laplacian. For ensembles of nonidentical oscillators without common noise, the incoherent state R(t)=0 is attractive and the average order parameter increases nonlinearly with the strength of common noise [Pimenova 2016].

How will that change with the number of influencers and followers?
From the analysis, the effect of coherence resonance driven by noise can be seen from the analysis of a single influencer. What is the effect of having more influencers?
How the coherence resonance driven by noise effect depends on the number of influencers and followers? A graph showing how the optimal q changes as a function of the number of influencers can be nice.

The question is certainly interesting. We expect that the coherence resonance will deteriorate with the diameter of network. In random influencer networks the diameter depends weakly on the number of influencers so we expect the effect to be robust. The optimal q (of order one) does not depend on the number of influencers. We have included another example in Figure F1 with a large number of influencers in the Supplemental Materials and discuss the effects of noise in Section C.

In sec 2.3.2. Slow fast dynamics, Eq. (16) is valid for D_0=0 and large partitioning, these assumptions should be stated in the methods section as well.

The effects of D0 and gamma0 are qualitatively and quantitatively similar (lines 117, 304-310) but Eqs. (12,16) are only exact for D0=0 and large partitions (line 297). In fact, heterogeneity and noise are exactly equivalent if the noise is Cauchy (line 310, new reference included [Toenjes 2020]).

In line 157, Under what assumption the field h_σ is constant? When will that be valid? Constant with respect to what?

At the fast time scale the fields h_k change slowly. The slow fast approximation assumes the h_k are constant (in time) at the fast time scale and the average field of an influencer follows the h_k adiabatically at the slow time scale (line 335-337).

What is the assumption on the noise strength for the followers, D_0?

The noise D_0 in the followers needs to be much smaller than D in the influencers (lines 120-122). We used D_0~Dx10^-3. The D_n may be heterogeneous.

Is there a threshold for D_0 for which you start to see the synchronization effect? Does the effect break down for high noise?

There is threshold depending on \Lambda (see Suppl. Fig. C1). We have included a plot of the resonance curves for different follower heterogeneities in the Supplementary Material Section C.

What is the optimal q for D_0 non-zero when there is no frequency heterogeneity?

Frequency heterogeneity and noise in the followers have the same effect. Equation (26) describes all solutions for an ideal influencer network on the synchronization manifold with gamma0 instead of D0. The optimal q is always close to 1.

And the interaction between them through the followers in both groups?

The average force G_k the influencer k exerts on its followers is a function of the weighted mean fields of all partitions connected to that influencer. The forces of different influencers are coupled through common followers. Eqs. (4,5) show this explicitly.

Is there an assumption on the number of influencers for which this effect is apparent?
Will it be enough to add noise only to one of the influencers to see this effect?

It is not enough to add optimal noise to one influencer only. Synchronization through influencers with non-optimal noise is poor (lines 103-108).

How do these results depend on the initial condition of the model?

Noise and small coupling strength make the effect independent of the initial phase distribution. We have clarified this point now (line 112). For large coupling the model may display multistability (line 227).

Questions and remarks about the simulations: Figure 1:
What is the difference in the parameters between the two figures?

We discuss the simulation parameters for Figure 1 in the Methods (lines 248-257). The

difference is a factor of 10 in the time scale separation from ~20 to ~200.

How many nodes are connected to both influencers a and b?

The three partitions in Fig.1 are of equal size $|P_1|=|P_2|=|P_3|=300$. This number is now in the caption of Fig.1 and we have also included it in Methods.

How do you determine the time T?

We estimate the transient time from simulations of the mean field ODEs (Supplemental Material Fig. D1). The time $T=10^4$ is 100 times longer than the transient.

Figure 2: Which nodes are considered as influencers and which ones are the followers? How do you determine the value of the noise for each node?

We describe the details of the simulation of Fig. 2 in Methods (lines 259-270). The influencers in the C. Elegans network and the scale free network are chosen as the K-largest degree nodes (K=15, K=5). The noise for followers is then chosen to display the most pronounced coherence resonance effect when the noise in the influencers is varied.

How does R(q) look like for these networks?

We show R(t) for small, optimal and large values of q in Fig. 2. Qualitatively the resonance curves for these networks look similar to the example in Fig.1 lower left panel.

What did we learn from these examples? Why should I expect that the optimal q is the same for these different configurations? Is this value universal?

We have shown and argued that the optimal q is always approximately one, i.e. when the time scale of the frequency gap and the time scale of the phase diffusion are comparable. Under this condition the response of the average influencer forces to the partition mean fields is optimal (Methods 2.4.2). The examples in Fig. 2 represent influencer networks with non-trivial topologies, e.g. scale free degree distribution, directional coupling and interaction between followers, demonstrating that the effect of coherence resonance can also be observed in complex networks.

Minor comments: Definition of the model – some parameters in the model are not well-defined. The matrix W_{nm} can the weight be negative? What are the range of values for possible weights?

We assume positive W_{mn} (line 56). Because of normalization μ_n the range of the W_{mn} is not very important. To ensure that the effect is robust against weak coupling between followers, we allow small follower interactions $W_{nm}=0.01$ compared to the connections $W_{km}=1$ to influencers.

It is not clear whether you consider uniform diffusion coefficient, D_i or does it change between nodes? In Eq. (1) the value is different for each node, but in the paragraph after it, a constant D is considered without stating it.

Different noise strengths are allowed, and indeed needed as the optimal noise in the influencers is much larger than in the followers. We have corrected the notation in line 69.

The same for the noise strength, q, also it is not defined properly in the main text.
$\Delta\omega$ Is not defined. Should that be γ_0?

We apologize. $\Delta\omega$ is the difference in natural frequencies between the influencers and the average frequency of the followers. We now mention this in the text (line 79) and in the new Table of parameters in Methods. The effective noise strength q was defined in Table 1 and we now repeat the definition in the main text (line 82).

What is the frequency distribution of the influencers with respect to followers?

A moderate $\Delta\Omega$ is needed to observe the effect of coherence resonance. This frequency gap can be different for each influencer.

The shifts in the frequencies is not clear,

The effect of shear is a shift of frequency proportional to the coupling strength (line 66). Shear and coupling heterogeneity are generally present in networks of coupled limit cycle oscillators but often omitted in reduced phase models. Refs. [26,27] have shown that shear leads to an effective frequency dispersion in networks with heterogeneous coupling.

It is not clear what is assumed about the initial distribution of frequencies for the followers and influencers. A better explanation in the supplementary material stating all the distribution and the transformation properly is needed.

The followers frequency have a narrow distribution (heterogeneity \gamma_0). The difference in natural frequency between influencers and followers together with shear contributes to the dynamical frequency gap. We now discuss the natural frequencies in lines 76-80 and in the new tables of parameters in Methods.

The manuscript contains many typos. The authors must check the manuscript carefully.

We are grateful for the feedback. We have contracted a professional editor to check spelling, grammar and language.

Coherence resonance in influencer networks

Ralf Tönjes[1], Carlos E. Fiore[2] and Tiago Pereira[3,4]

[1] *Institute for Physics and Astronomy, University of Potsdam, Karl-Liebknecht-Str. 24, 14476 Potsdam, Germany*

[2] *Instituto de Física, Universidade de São Paulo, São Paulo, Brazil*

[3] *Instituto de Ciências Matemáticas e Computação, Universidade de São Paulo, São Carlos, São Paulo, Brazil*

[4] *Department of Mathematics, Imperial College London, SW7 2AZ, London, UK*

Complex networks are abundant in nature and many share an important structural property: they contain a few nodes that are abnormally highly connected (hubs). Some of these hubs are called 'influencers' because they couple strongly to the network and play fundamental dynamical and structural roles. Strikingly, despite the abundance of networks with influencers, little is known about their response to stochastic forcing. Here, for oscillatory dynamics on influencer networks, we show that subjecting influencers to an optimal intensity of noise can result in enhanced network synchronization. This new network dynamical effect, which we call 'coherence resonance in influencer networks', emerges from a synergy between network structure and stochasticity and is highly nonlinear, vanishing when the noise is too weak or too strong. Our results reveal that the influencer backbone can sharply increase the dynamical response in complex systems of coupled oscillators.

A central discovery in network science is that a small group of highly connected hubs can couple to the network more strongly than their peers and greatly influence the network behavior [1–6]. Examples of network influencers can be found in neuroscience (e.g., normal and aberrant synaptic connectivity [7–10]), political opinions (e.g., election blogging [11] or social networks), and man-made scale-free networks (e.g., the internet [1]). Surprisingly, the presence of such influencers makes synchronization of deterministic network dynamics more difficult because networks with influencers require stronger coupling than homogenous networks [12, 13]; indeed, in many situations, synchronization of influencer networks cannot be achieved at all [14, 15]. This observation is all the more remarkable because synchronization plays a fundamental role in regulating network function [16, 17] and is mediated predominantly through influencers [7, 18, 19]. This raises a crucial question: why have many real-world networks evolved to contain influencers when they appear to be detrimental to the network dynamics, at least at face value?

In the present study, we show that stochastic forcing of influencers can lead to an optimal collective network response. Strikingly, introduction of noise synergizes with the network structure to create collective oscillations that become optimal at a given noise strength in the influencers. This phenomenon emerges in two steps. First, the network acts as a nonlinear filter for the stochastic influencer dynamics, and at an optimal noise strength, the influencers induce synchronization in the nodes directly connected to them. Second, different parts of the network develop macroscopic dynamics and interact indirectly through the influencers. We develop an adiabatic theory to uncover this macroscopic interaction law and show that it mediates the emergence of global col-

lective oscillations. When the noise in the influencers is either too weak or too strong, the coupling vanishes. Interestingly, at a macroscopic level, the interaction between different parts of the network can be described by a hyper-graph. The term coherence resonance was originally coined for an optimal response of noise-induced oscillations in excitable systems [20] and homogeneous networks or all-to-all coupling [21]. Coherence resonance was also observed in non-excitable systems near bifurcation [22] and between two coupled oscillators [23]. However, a network dependent mechanism for coherence resonance has not previously been described.

Dynamics on influencer networks. We refer to a network where most nodes couple predominantly to a small number of influencers as an 'influencer network', and refer to the remaining nodes as 'followers' (Figure 1). Given a network weight coupling matrix $W_{nm} \geq 0$, which is nonzero if node n receives a link from node m, the intensity μ_n is the total coupling weight received by the nth node. As generic oscillatory dynamics, we consider a network of oscillators

$$\dot{\vartheta}_n = \omega_n + \frac{\lambda_n}{\mu_n} \sum_{m=1}^{N} W_{nm} g(\vartheta_m, \vartheta_n) + \sqrt{2D_n}\xi_n. \quad (1)$$

Here, ω_n is the natural frequency of node n, which couples with strength λ_n to the weighted mean of the coupling functions g to neighboring nodes m. A table of parameters and their function is provided in Methods 2.2. The coupling

$$g(\vartheta_m, \vartheta_n) = \sin(\vartheta_m - \vartheta_n - \alpha) + c_0$$

is generic for weakly coupled, nearly identical oscillators [24, 25]. The parameter α is called phase frustration and the bias c_0 is due to shear, an amplitude dependence of the frequency [26, 27]. The effect of shear is a shift in the average frequency proportional

to the coupling strength. Phase equations with this form of coupling g are known as the Kuramoto-Sakaguchi model [28, 29] and are widely applied accross scientific disciplines [24–29]. In addition, each term $\sqrt{2D_n}\xi_n$ denotes uncorrelated Gaussian white noise of strengths D_n. In many studies, the coupling strength λ_n to the local mean-field is chosen to be uniform, in which case the coupling is called normalized. In real-world and experimental systems, though, coupling may be heterogenous and hubs can couple more strongly to the network [5, 18]. We model this coupling as

$$\lambda_n = \begin{cases} \beta_n \lambda_0 & \text{for influencers} \\ \lambda_0 & \text{for followers.} \end{cases}$$

For simplicity, throughout this exposition we consider the coupling intensity $\beta_n = \beta$, the noise strength $D_n = D$, and the frequency $\omega_n = \omega$, to be identical for all influencers. For the followers we assume a Lorentzian frequency distribution with mean frequency ω_0 and width γ_0. Noise is of identical strength $D_n = D_0$ in all followers. We denote $\Delta\omega = \omega - \omega_0$ the average gap in natural frequencies between influencers and followers. In Methods 2.1 we show how Eq. (1) can be recast in terms of dimensionless effective parameters shown in Table 1. These effective parameters, and in particular the influencer effective noise strength $q = D/\Delta\Omega$, play key roles in the collective dynamics of the system. The dynamical frequency gap $\Delta\Omega/\lambda_0$ leads to a time scale separation between the dynamics of the followers and the influencers. A coupling intensity β of comparable but smaller magnitude leads to an effective coupling strength Λ close to one for which the effect of coherence resonance is most pronounced. We note that the dynamical frequency gap needs to be large in units of λ_0, but it can be small in natural time units. In the Supplementary Material Section A, we present an

Parameter	Meaning	range
$\Delta\Omega/\lambda_0 = \Delta\omega/\lambda_0 + (\beta - 1)c_0$	dynamical frequency gap	$\Delta\Omega/\lambda_0 \gg 1$
$\Lambda = \beta\lambda_0/\Delta\Omega$	dimensionless coupling strength	$\Lambda < 1$
$q = D/\Delta\Omega$	influencer effective noise strength	$q = O(1)$
D_0/λ_0	followers effective noise strength	$D_0/\lambda_0 \ll 1$
γ_0/λ_0	followers frequency heterogeneity	$\gamma_0/\lambda_0 \ll$

Table 1: **Effective dynamical parameters in influencer networks**. We obtain these parameters as described in Methods 2.3. These are key parameters in the description of coherence resonance and the optimal noise strength in the influencers.

example of the transformation for realistic parameters in Eq. (1) to effective parameters.

We divide the followers into partitions P_σ of nodes connected to the same set of influencers. In Figure 1, we show an influencer network with two influencers (a and b) and three partitions of followers (σ=1,2,3), which are connected to influencers a, b, or both (see additional examples in Supplementary Material Section B). To capture the collective dynamics in each partition σ, we introduce the complex mean-fields

$$Z_\sigma(t) = \frac{1}{|P_\sigma|} \sum_{n \in P_\sigma} e^{i\vartheta_n(t)} \tag{2}$$

The modulo of the complex mean-field $R_\sigma = |Z_\sigma|$ is the partition order parameter; that is, $R_\sigma = 0$ for incoherent, uniformly distributed phases and $R_\sigma = 1$ in full synchrony. Similarly, the global mean-field Z and order parameter R are defined by summing over all followers in the network.

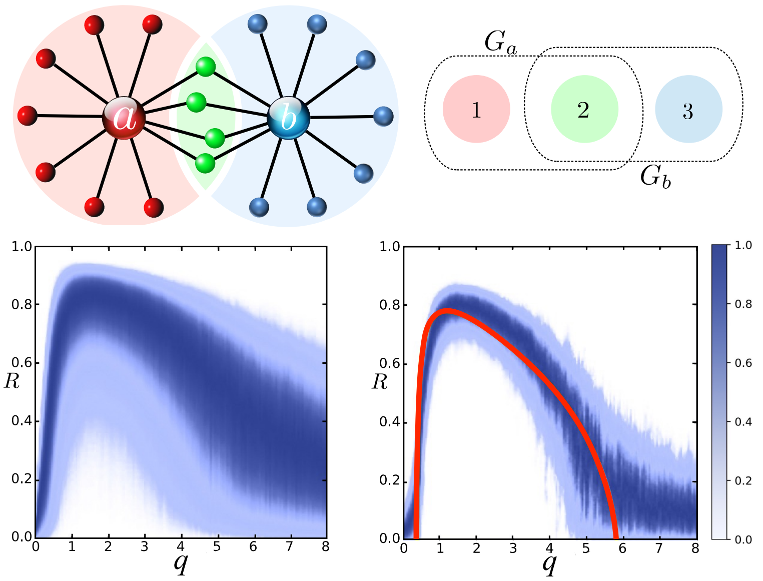

Figure 1: **Coherence resonance in an influencer network.** We show the distribution of the order parameter R versus the effective diffusion q in the influencers. Influencers a and b are hubs that couple strongly to the network, and all other nodes are regarded as followers (upper left schematic). Three distinct partitions of followers are shown in red, blue, and green, which connect to influencer a, b, and both a and b respectively. Followers are subject to noise with diffusion constant D_0, whereas influencers are subject to independent noise with diffusion constant D. In our simulation, each partition has 300 followers. For each value of effective noise strength q in the influencers, we plot the density of the global order parameter R on a color scale from 0 (white) to the maximum value (blue). At an optimal noise strength, the mean value of the global order parameter reaches a maximum, revealing the coherence resonance effect. The solid red line (lower right panel) is our theoretical prediction. Our theory predicts that the interactions of the macroscopic mean-fields take place in a hyper-graph mediated by the coupling functions G_a and G_b (upper right schematic) see Methods 2.4.4.

1 Results

With deterministic influencers where $q = 0$, and when $|\Lambda| < 1$, the influencers cannot frequency lock to the followers. Synchronization of the followers through the influencer backbone is poor and counteracted by noise and frequency heterogeneity in the followers. Our results show that by setting a weak noise strength or frequency heterogeneity in the followers and by changing the effective noise q in the influencers, synchronization of the whole network increases, reaches a maximum, and then decreases.

We numerically integrate our model Eq. (1) in dimensionless units (Table 1) for the network with two influencers (as shown in Figure 1) with 300 identical followers in each partition, and a small fixed noise strength in the followers. By changing the noise strength in the influencers, we then obtain the distribution of the order parameter R as a function of q. After a transient, the order parameter is independent of the initial conditions. At an optimal noise strength, R reaches its maximum (in expected value), as shown in Figure 1 for $\Delta\Omega/\lambda_0 = 18$ (left panel) and for $\Delta\Omega/\lambda_0 = 198$ (right panel). The solid line is a theoretical prediction in the thermodynamic limit for heterogeneous followers using a slow-fast approximation. Frequency heterogeneity and noise in the followers have qualitatively and quantitatively the same desynchronizing effect. Optimal synchronization of the whole network is predicted theoretically and achieved in all simulations for an effective noise strength $q \approx 1$ in the influencers (see details in Methods 2.4.2). Our mean-field analysis predicts that the effect of coherence resonance is only observed for very small frequency heterogeneity or noise in the followers, below

a threshold that depends on Λ (Supplementary Material Section C).

In Figure 2, we show the time series of the order parameter R for two complex and real-world networks; the lower row represents a directed neural network in the model organism Caenorhabditis elegans, and the upper row represents a scale-free network. We assign the role of influencers to the K most strongly connected nodes and use a weighted connectivity matrix $W_{nm} = 1$ for all connections from or to an influencer, and $W_{nm} = 0.01$ for all other connections. For small effective noise q in the influencers (Figure 2, 'q weak'), the order parameter fluctuates at a low level. When $q = 1$ ('q optimal'), the order parameter fluctuates around a value close to 1, revealing coherent collective oscillations. Finally, when q is large ('q strong'), the order parameter decreases again, revealing the loss of synchrony. All parameters for the simulation and numerical scheme can be found in Methods 2.2. In the Supplementary Material Section D, we show three additional examples of coherence resonance in influencer networks; with 3 influencers, a random network with 100 influences, and the third is a network of linked political blogs.

Stochastic forcing by a single influencer. Let us consider a single influencer. When its followers are asynchronous, the sinusoidal contributions in the sum of the coupling functions for that influencer average out, and the influencer phase is effectively decoupled from the followers. The influencer is independent of the network and acts as a common stochastic force on the followers connected to it. The additive noise in the influencer enters the dynamics of its followers multiplicatively through the coupling function. That is, the network acts as a nonlinear filter for the noise in the in-

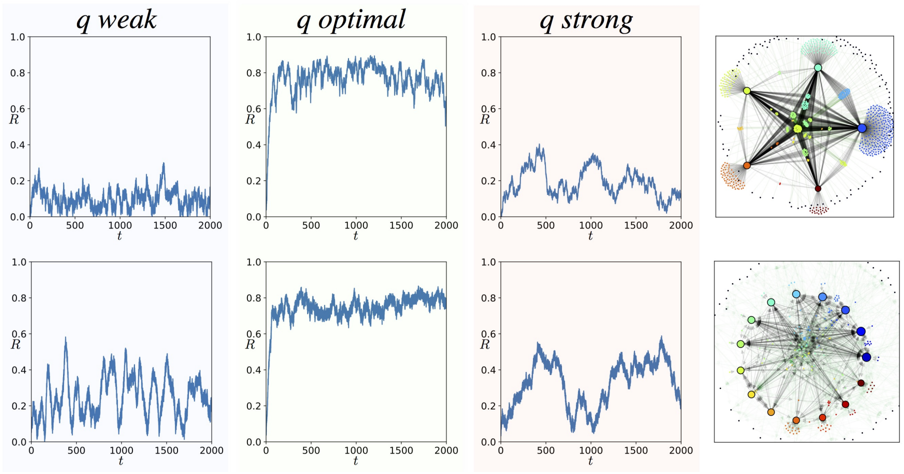

Figure 2: **Coherence resonance of the order parameter across multiple networks**. Columns 1-3 show the time series of the order parameter R for three values of noise strength in the influencers (weak $q = 0.1$, optimal $q = 1$, and strong q=10). Column 4 shows the corresponding networks. Upper row, scale-free network with exponent 2 [1]; lower row, C. elegans directed neural network [3]. See Methods 2.3 for further details. Additional examples can be found in the Supplementary Material Section D.

fluencers. We can show that the effective diffusion constant of the integrated stochastic forcing in the followers, a proxy for the noise strength, attains a maximum at an optimal effective diffusion when

$$q_{\text{opt}} = 1\,. \tag{3}$$

We present more detailed calculations in Methods 2.4.2 and in the Supplementary Material Section E. When oscillations are driven by common multiplicative noise, the effect of noise-induced synchronization can be observed [30]. As the common noise intensity is increased the oscillators synchronize faster. This suggests that at this optimal value of q the incoherent state will be most unstable. However, behavior of the

noise transfer does not explain the synchronization between different partition mean-fields. Because this synchronization requires studying macroscopic dynamics of R far from zero, our next goal is to uncover the interaction function between the mean-fields in different partitions.

Mean-field dynamics of partitions takes place in a hyper-network. For simplicity, we provide an analysis of the influencer network shown in Figure 1. Mean-field equations for mixed repulsive and attractive coupling or intra- and inter-partition interactions in the followers can be generalized from these results. In Methods 2.4.1, we show that assuming (i) large partition sizes $|P_\sigma| \gg 1$, (ii) large dynamical frequency gap $\Delta\Omega/\lambda_0 \gg 1$, and (iii) noise free followers $D_0 = 0$ with frequency heterogeneity γ_0, it is possible to derive averaged dynamics of the partition mean-fields Z_σ in an adiabatic approximation. The resulting deterministic equations have the structure of a hyper-network. In our example, the governing equations are

$$\dot{Z}_1 = F(Z_1, G_a), \quad \dot{Z}_2 = F\left(Z_2, \frac{1}{2}G_a + \frac{1}{2}G_b\right) \quad \text{and} \quad \dot{Z}_3 = F(Z_3, G_b) \tag{4}$$

where the coupling functions are

$$G_a = G(w_{a1}Z_1 + w_{a2}Z_2) \quad \text{and} \quad G_b = G(w_{b2}Z_2 + w_{b3}Z_3). \tag{5}$$

F describes a Riccati force (see Methods 2.4.1). The weights $w_{k\sigma}$ with $\sum_\sigma w_{k\sigma} = 1$ denote the relative size of partition σ among all followers of influencer k. Note that, while in the microscopic description the connections between nodes are pairwise, at the level of mean-fields, edges represented by a coupling function G_k for each influencer can connect multiple partitions of followers. Thus, the mean-field interaction between different parts of the network is described by a hyper-graph.

The interaction functions G_a and G_b can be determined analytically; they depend on Λ and q, are maximal at an optimal noise strength, and vanish at critical values of q. That is, at weak or strong noise in the influencers, the hyper-network interactions vanish, revealing the highly nonlinear nature of the phenomenon. In particular, this means that the macroscopic fields will not interact in the strong noise limit. We derive the analytic expressions for the coupling functions $G_k(Z; \Lambda, q)$ in Methods section 2.4.2.

Global synchronization and resonance. When influencers have equal parameters q and Λ the synchronization manifold $Z_\sigma = Z$ is invariant under (4) and (5) and stable for phase-attractive coupling. Hence, the macroscopic fields synchronize and we can explain the global coherence resonance by restricting the analysis to this invariant subspace. Our mean-field theory predicts both effects: the coherence resonance of the partition order parameters and phase synchronization of the partition mean-fields as shown in Methods 2.4.4. The solid line in Figure 1 (right) is the stationary average order parameter predicted by our theory in the infinite time-scale separation limit and with frequency heterogeneity $\gamma_0/\lambda_0 = 0.02$ in the followers. The predicted values agree with simulations of the finite size network, large dynamical frequency gap $\Delta\Omega/\lambda_0 = 198$ and identical followers with noise $D_0/\lambda_0 = 0.02$.

Summary and discussions. We have found a new effect induced by a synergy between noise and network structure to generate a transition towards a synchronization that would not be possible in the absence of noise. The key element for this effect is the existence of influencers – a group of hubs that couple strongly and connect different

parts of a network. Although deterministic network parameters prevent synchronization, we show that an optimal noise strength in the influencers can induce and mediate synchronization. The mechanism for this coherence resonance in influencer networks is different from the known effect of coherence resonance in homogeneous networks with excitatory dynamics, where noise simply excites oscillations [20, 23, 31]. At the macroscopic level, the interaction between different parts of the network is indirect and takes place on an emerging hyper-network, thus changing the interaction structure from the microscopic level. Such higher order interactions have previously been conjectured and reported in neuronal data recordings [32]. Our findings suggest that the emergent order in complex systems could be controlled by regulating the noise in only a few key nodes.

2 Methods

2.1 Canonical form

To bring the Eq. (1) into a dimensionless form with effective parameters given in Table 1, we change the time scale to units of λ_0^{-1} and add the frequency shift from the bias in the coupling function to the natural frequencies of the oscillators, i.e. $\omega_n \mapsto \omega_n + \lambda_n c_0$ and $g(\vartheta_m, \vartheta_n) \to \sin(\vartheta_m - \vartheta_n - \alpha)$. The difference between the average follower frequency and the frequency of an influencer, both including the frequency shift from the coupling bias, is the dynamical frequency gap $\Delta\Omega/\lambda_0$ (in units of λ_0). Observing the invariance of the phase equations under a global phase shift, i.e. $\vartheta \to \vartheta - \omega_0 t$,

we can go into a co-rotating reference frame where the average follower frequency is zero. The deviations of the follower frequencies from their mean frequency ω_0 may be written as $\gamma_0 \nu_n$ where the ν_n are taken from some standard distribution with mean zero and the factor $\gamma_0 \geq 0$ characterizes the frequency heterogeneity. Then the phase equations for the followers in the new time units and co-rotating reference frame are

$$\dot{\vartheta}_n = \frac{\gamma_0}{\lambda_0}\nu_n + \frac{\lambda_n}{\lambda_0}\frac{1}{\mu_n}\sum_k W_{nk}\sin(\vartheta_k - \vartheta_n - \alpha) + \sqrt{2D_n/\lambda_0}\tilde{\xi}_n \tag{6}$$

and for the influencers with phases ψ_k

$$\dot{\psi}_k = \frac{\Delta\Omega_k}{\lambda_0}\left(1 + \Lambda_k \frac{1}{\mu_k}\sum_m W_{km}\sin(\vartheta_m - \psi_k - \alpha)\right) + \sqrt{2q_k\frac{\Delta\Omega_k}{\lambda_0}}\tilde{\xi}_k. \tag{7}$$

Here, $\Lambda_k = \lambda_k/\Delta\Omega_k$ is the ratio between the coupling strength and the frequency of the influencer. In the noise free case, phase locking is only possible for $|\Lambda_k| > 1$. Changing Λ can lead to a discontinuous, explosive synchronization [12, 33]. The term $\tilde{\xi}_m$ are independent white noise with $\langle\tilde{\xi}_m(t)\tilde{\xi}_n(t')\rangle = \delta_{mn}\delta(t - t')$ in the new units of time and q_k is the effective noise strength in the influencers on the fast time scale $\Delta\Omega_k/\lambda_0$. In the Supplementary Material Section A, we provide examples of such rescaling.

2.2 Parameters and their meaning

In Table 2, we present the main parameters that naturally appear in the phase model Eq. (1) and give rise to effective parameters, as shown in Table 1 in the main text. The main parameters in our mean-field analysis are shown in Table 3.

Parameter	Meaning
ω_n	isolated frequency of the nth oscillator;
	set as $\omega_n = \omega_0 + \gamma_0 \nu_n$ for followers and ω for influencers
ω_0	mean frequency of the followers
$\gamma_0 \nu_n$	frequency deviation $\omega_n - \omega_0$ of the nth follower
γ_0	scale parameter of follower frequency distribution
$\Delta\omega$	gap $(\omega - \omega_0)$ between influencer and average follower frequency
W_{nm}	nonnegative matrix of connection weights
μ_n	connection intensity ($\mu_n = \sum_m W_{nm}$)
λ_n	coupling strength of the nth oscillator;
	λ_0 for followers and $\beta\lambda_0$ for influencers
β	coupling intensity for influencers
D_n	diffusion constant set as D for influencers and D_0 for followers
α	phase frustration in the coupling function g
c_0	shear parameter in the coupling function g

Table 2: **Parameters in the model presented in Eq. (1) of the main manuscript**.

2.3 Simulation and Parameters

In our analysis and our simulations, we use the transformed, dimensionless canonical form (6) and (7) of the phase equations (1). Existing connections in the network from and to the influencers are given the weight $W_{mn} = 1$ and connections between followers $W_{nm} = 0.01$. The followers couple to their neighbors with strength $\lambda_n = \lambda_0$

Parameter	Meaning
P_σ	follower partitions according to the influencers they connect to
I_σ	set of influencers of a partition σ
Z_σ	complex mean-field of partition σ (order parameter $R_\sigma = \lvert Z_\sigma \rvert$)
G_k	coupling function between mean-fields mediated by influencer k
$w_{k\sigma}$	coupling weights of the mean-field between influencer k and partition σ
F	Ricatti vector field see Eq. (12)
h_σ, h_k	forcing on oscillators in partition σ and on influencer k
H_σ	average of h_σ obtained from adiabatic mean-field approximation

Table 3: **Parameters of the mean-field analysis presented in Eq. (4).**

and more strongly to the influencers with $\lambda_k = \beta\lambda_0$. The phase frustration in the coupling function is set to $\alpha = -0.1$. The frequency deviations ν_n of the followers are drawn from a Cauchy distribution $p(\nu) = 1/\pi(\nu^2+1)$ and multiplied by γ_0/λ_0. Thus, frequency heterogeneity and noise strength in the followers are given by γ_0/λ_0 and D_0/λ_0, respectively. We chose $\beta_k = \beta$, $\lambda_k = \beta\lambda_0$ and $D_k = D$ for all influencers, so that $\Delta\Omega/\lambda_0$, Λ and q are identical for all influencers. We integrate the Langevin equations of the phases with an Euler-Maruyama scheme and small time steps $dt = 5 \cdot 10^{-4}$ because of the large time scale separation $\Delta\Omega/\lambda_0 \gg 1$.

Parameters of Figure 1: The network structure is a pure influencer network without connections between followers or between influencers. We simulate 300 identical followers $\gamma_0/\lambda_0 = 0$ in each of the three partitions with small independent noise

$D_0/\lambda_0 = 0.02$. In the lower left panel we have $\beta = 10$, a dynamical frequency gap of $\Delta\Omega/\lambda_0 = 18$ and an effective influencer coupling strength $\Lambda = 10/18$. In the lower right panel $\beta = 100$, $\Delta\Omega/\lambda_0 = 198$ and $\Lambda = 100/198$. For each value of the effective influencer noise strength $q = D/\Delta\Omega$ we record a histogram of the order parameter over $T = 10^4$ time units, which is much longer than the relaxation time of R. The theoretical prediction, the solid line in the lower right panel, is for noiseless followers $D_0 = 0$ and $\gamma_0/\lambda_0 = 0.02$.

Parameters of Figure 2: For the *C. elegans* directed neuronal network [3], we choose the top $K = 15$ out-degree nodes as influencers. All nodes with zero in-degree have been removed, resulting in a network with $N = 268$ nodes. Connections between followers are given the weight $W_{nm} = 0.01$. We simulate identical followers $\gamma_0/\lambda_0 = 0$ with small independent noise $D_0/\lambda_0 = 0.02$. The dynamical frequency gap between followers and influencers is $\Delta\Omega/\lambda_0 = 18$ and the effective coupling strength in the influencers is $\Lambda = 10/18$. Shown are three time series of the network order parameter for small, optimal, and large noise strength q in the influencers. The undirected scale-free network with exponent 2 is the largest connected component of a network generated via a configurational algorithm [1] without self loops or double edges. We chose the top 5 degree nodes as influencers. The other parameters are the same as in the *C. elegans* neuronal network.

2.4 Mean-field dynamics

2.4.1 Mean-field dynamics in influencer networks

We have developed a mean-field theory for undirected influencer networks with connections exclusively between influencers and followers, as shown in Figure 1. This theory can be generalized to more complex configurations, heterogenous influencers, directed, attractive, or repulsive coupling between followers and influencers, within partitions or between different partitions. While these generalizations may lead to more complex dynamic behavior, the mechanism for the coherence resonance is apparent in the simplest model.

We consider the network as a union of a set P of followers and a set I of influencers. The vertices n connected to an influencer k are elements $n \in P_k$ of the periphery of the influencer k. Intersections of the sets P_k form equivalence classes or partitions P_σ of followers that are connected to the same subsets I_σ of influencers such as in Fig.1 all followers connected to influencer a or b or to both influencers. The phases of the oscillators are encoded as complex variables $z_n = \exp(i\vartheta_n)$ for the followers and $z_k = \exp(i\psi_k)$ for the influencers. The dynamics can be formulated in terms of partition averages and averages over the influencers of these partitions

$$Z_\sigma = \frac{1}{|P_\sigma|} \sum_{n \in P_\sigma} z_n \tag{8}$$

$$h_\sigma = \frac{e^{-i\alpha}}{2i} \frac{1}{|I_\sigma|} \sum_{k \in I_\sigma} z_k \tag{9}$$

$$h_k = \frac{e^{-i\alpha}}{2i} \frac{1}{|P_k|} \sum_{n \in P_k} z_n = \frac{e^{-i\alpha}}{2i} \sum_\sigma w_{k\sigma} Z_\sigma. \tag{10}$$

Here, h_σ and h_k are the forces acting on the followers in partition σ and on the influencer k. The weight $w_{k\sigma}$ is the relative size of partition σ in the periphery of an influencer k; that is, $w_{k\sigma} = |P_\sigma|/|P_k|$ when $P_\sigma \subseteq P_k$ or $w_{k\sigma} = 0$ otherwise. Thus, the phase dynamics (6) and (7) can be written in complex form as

$$\begin{aligned} \dot{z}_n &= iz_n\left(\bar{h}_\sigma z_n + \frac{\gamma_0}{\lambda_0}\nu_n + h_\sigma \bar{z}_n\right) + iz_n\sqrt{2D_0/\lambda_0}\xi_n(t), \qquad n \in P_\sigma \\ \dot{z}_k &= iz_k\frac{\Delta\Omega_k}{\lambda_0}\left(\Lambda_k\bar{h}_k z_k + 1 + \Lambda_k h_k \bar{z}_k\right) + iz_k\sqrt{2D_k/\lambda_0}\xi_k(t) \end{aligned} \tag{11}$$

The first reduction of model complexity is via the Ott-Antonsen approach [34] for followers without Gaussian white noise but frequency heterogeneity γ_0/λ_0 with Cauchy-distributed frequency deviations ν_n. In the thermodynamic limit $|P_\sigma| \to \infty$ (keeping the ratios $w_{k\sigma}$ of the partition sizes constant) there exists a globally attractive invariant manifold on which the mean-fields Z_σ evolve by a complex Riccati equation as

$$\dot{Z}_\sigma = i\left(\bar{h}_\sigma Z_\sigma^2 + i\frac{\gamma_0}{\lambda_0}Z_\sigma + h_\sigma\right) = F(Z_\sigma, h_\sigma). \tag{12}$$

For large partition sizes, Eqs. (8-12) provide a good description of the system dynamics, including an accurate description of the fluctuations of the mean-fields (see the Supplementary Materials Section F). The effect of small noise D_0/λ_0 in the followers is comparable to the effect of frequency heterogeneity γ_0/λ_0. For small white noise, the Ott-Antonsen manifold is no longer invariant but one can derive a hierarchy of corrections to the dynamics (12) in increasing orders of the noise strength. To the zeroth order the effects of frequency heterogeneity and noise are identical [35]. In fact, if the noise in the followers is white Cauchy noise, the equivalence of noise and frequency heterogeneity is exact [36].

2.4.2 Slow-fast dynamics

If there is a large dynamical frequency gap $\Delta\Omega_k/\lambda_0 \gg 1$ between the followers and an influencer, oscillators in the follower group experience an average force from the fast influencer. Conversely, if the followers are desynchronized, the mean-field of the followers vanishes and the influencer phases perform a drift diffusion process on the circle

$$\dot{\psi}_k = \frac{\Delta\Omega_k}{\lambda_0} + \sqrt{2D_k/\lambda_0}\tilde{\xi}_k(t) \tag{13}$$

whereas the followers connected to only that influencer experience a stochastic forcing by the influencer phase

$$\dot{\vartheta}_n = \frac{\gamma_0}{\lambda_0}\nu_n + \sin(\psi_k - \vartheta_n - \alpha) + \sqrt{2D_0/\lambda_0}\tilde{\xi}_n(t). \tag{14}$$

This forcing is multiplicative since $\sin(\psi_k - \vartheta_n - \alpha) = s_k \cos\vartheta_n - c_k \sin\vartheta_n$ with two uncorrelated but not independent random processes $s_k(t) = \sin(\psi_k - \alpha)$ and $c_k(t) = \cos(\psi_k - \alpha)$. The diffusion constants D_s and D_c for these stochastic forces quantify an effective noise strength and can be calculated as the integral of the respective autocorrelation functions [37]

$$\frac{D_s}{\lambda_0} = \frac{D_c}{\lambda_0} = \frac{\lambda_0}{2}\frac{D_k}{\Delta\Omega_k^2 + D_k^2} = \frac{\lambda_0}{2\Delta\Omega_k}\frac{q_k}{1+q_k^2}. \tag{15}$$

We present the details in the Supplementary Material Section E. By changing the noise strength D_k, the effective noise strengths D_s and D_c have a maximum at $D_k = \Delta\Omega_k$ or $q_k = D_k/\Delta\Omega_k = 1$. At this noise value, and for incoherent followers, the effect of noise-induced synchronization [30] is expected to be strongest. As the amplitudes of s_k and c_k are bounded, when D_k or the time scale separation $\Delta\Omega/\lambda_0$ are further

increased the effective noise strengths go to zero.

For $\Delta\Omega/\lambda_0 \gg 1$, the system has slow and fast dynamics and we can replace the influencer phases z_k contributing to the force fields h_σ (9) in each partition σ by the expected values G_k of z_k subject to Langevin equation (11). On the fast time scale of the influencers, the fields h_k are changing very slowly and can assumed to be constant for the calculation of the G_k. In this averaged dynamics, the influencers create an average force H_σ that follows the partition mean-fields adiabatically. The slow dynamics of the partition mean-fields is thus given as

$$\dot{Z}_\sigma = i\left(\bar{H}_\sigma Z_\sigma^2 + i\frac{\gamma_0}{\lambda_0}Z_\sigma + H_\sigma\right) = F(Z_\sigma, H_\sigma) \tag{16}$$

$$H_\sigma = \frac{e^{-i\alpha}}{2i}\frac{1}{|I_\sigma|}\sum_{k\in I_\sigma} G_k\left(\sum_{\sigma'} w_{k\sigma'}Z_{\sigma'};\ \Lambda_k, q_k\right). \tag{17}$$

This corresponds to a hyper-graph with partitions σ as nodes and coupling functions G_k for each edge k of the hyper-graph. General setups can be considered as well, with intra and inter-partition coupling and connections between influencers. The absence of such connections shows that the synchronization is indeed a noise-induced effect.

2.4.3 Mean-field of the fast influencers

The Langevin equation (11) for z_k with constant fields h_k is indeed a complex formulation of the noisy Adler equation [37]

$$\dot{\psi} = \frac{\Delta\Omega}{\lambda_0}\left(1 + 2\Lambda\mathrm{Im}\left[he^{-i\psi}\right]\right) + \sqrt{2D/\lambda_0}\xi(t). \tag{18}$$

The expected value G of $z = \exp(i\psi)$ is the first circular moment of the stationary distribution that has an expression as a continued fraction [37] and evaluates to a ratio

of confluent hypergeometric limit functions ${}_0F_1(o,x)$ [38]. It can be derived from the Fokker-Planck equation noting that the Fourier modes $p_k = \langle \exp(ik\psi) \rangle$ of the stationary distribution $p^{st}(\psi)$ are in a tridiagonal recurrence relation

$$0 = ik\Delta\Omega\left(\Lambda\bar{h}p_{k+1} + p_k + \Lambda h p_{k-1}\right) - Dk^2 p_k \tag{19}$$

which is solved by a continued fraction. Defining $q = D/\Delta\Omega$ and

$$s = \frac{|h|}{ih}, \quad o = 1 - i\frac{1}{q}, \quad \text{and} \quad x = \frac{q}{\Lambda|h|} \tag{20}$$

we have $G = p_1$ and

$$G = \frac{1}{o + \frac{x^{-2}}{(o+1) + \frac{x^{-2}}{(o+2)+\dots}}}\frac{1}{sx} = \frac{{}_0F_1(o+1, x^{-2})}{{}_0F_1(o, x^{-2})}\frac{1}{sox}. \tag{21}$$

2.4.4 Synchronization manifold and prediction of order parameter

If all influencers have the same effective noise strength $q = q_k = \frac{D_k}{\Delta\Omega_k}$ and the same effective coupling strength $\Lambda = \Lambda_k = \frac{\beta\lambda_0}{\Delta\Omega}$, the *synchronization manifold* where all partitions have identical mean-fields $Z_\sigma = Z = R^{i\Theta}$ is invariant under the averaged dynamics (16) and (17) on the hyper-graph and we can write

$$\dot{Z} = i\left(\bar{H}Z^2 + i\frac{\gamma_0}{\lambda_0}Z + H\right) \tag{22}$$

$$H = \frac{e^{-i\alpha}}{2i}G\left(Z;\ \Lambda, q\right) \tag{23}$$

where $G(Z;\ \Lambda, q)$ is defined as (21) with

$$h = \frac{e^{-i\alpha}}{2i}Z. \tag{24}$$

In particular, because of rotational symmetry, the dynamics of the amplitude $R = |Z|$ does not depend on the angle Θ of the mean-field

$$\dot{R} = \text{Re}\left[\frac{e^{-i\alpha}}{2} G(R;\ \Lambda, q)\right]\left(1 - R^2\right) - \frac{\gamma_0}{\lambda_0} R. \tag{25}$$

If the synchronization manifold is stable, the stable fixed points of this dynamics where $\dot{R} = 0$ approximate the average order parameter over all followers. From (25) we find that the level sets of the right-hand side of

$$\frac{\gamma_0}{\lambda_0} = \text{Re}\left[\frac{e^{-i\alpha}}{2} G(R;\ \Lambda, q)\right] \frac{1 - R^2}{R} \tag{26}$$

determine this average order parameter R for any given γ_0/λ_0 implicitly. We show this prediction for $\gamma_0/\lambda_0 = 0.02$ and $\Lambda = 0.56$ as a solid line in the lower right panel in Fig.2. Further resonance curves and maxima of R for different heterogeneities γ_0 and different Λ can be found in the Supplemental Material Section C.

Acknowledgements We would like to thank C. Sagastizábal, D. Eroglu, S. van Strien, D. Turaev, J. Lamb, and A. Pikovsky for enlightening discussions. This work was supported in parts by the DFG and FAPESP through the IRTG 1740/TRP 2015/50122-0, by the Center for Research in Mathematics Applied to Industry (FAPESP Cemeai grant 2013/07375-0) and grants 2015/04451-2, by the Royal Society London, CNPq grant 302836/2018-7, and by the Serrapilheira Institute (Grant No. Serra-1709-16124).

Data Availability. All relevant data are available from the corresponding author upon request.

Code Availability. Input files or sets of input parameters for Fortran as well as self-

developed Python codes are available from the corresponding author upon request.

Author contributions. RT and TP wrote the text and developed the theory. RT made the slow fast approximation and numerical solutions of the special functions. CEF made simulations of Figure 1. TP and RT made the Figures.

Competing interests. Authors declare no competing interests.

References

[1] Newman, M. E. J. *Networks: An Introducton* (Oxford University Press, Oxford, 2010).

[2] Xu, X.-K., Zhang, J. & Small, M. Rich-club connectivity dominates assortativity and transitivity of complex networks. *Phys. Rev. E* **82**, 046117 (2010).

[3] Watts, D. J. & Strogatz, S. H. Collective dynamics of small-worldnetworks. *Nature* **393**, 440–442 (1998).

[4] van den Heuvel, M. P. & Sporns, O. Network hubs in the human brain. *Trends Cogn. Sci.* **17**, 683–696 (2013).

[5] Song, S., Sjöström, P. J., Reigl, M., Nelson, S. & Chklovskii, D. B. Highly nonrandom features of synaptic connectivity in local cortical circuits. *PLoS Biol.* **3**, e68 (2005).

[6] Fulcher, B. D. & Fornito, A. A transcriptional signature of hub connectivity in the mouse connectome. *Proc. Natl. Acad. Sci. USA* **113**, 1435–1440 (2016).

[7] Bonifazi, P. *et al.* Gabaergic hub neurons orchestrate synchrony in developing hippocampal networks. *Science* **326**, 1419–1424 (2009).

[8] Schneidman, E., Berry, M. J., Segev, R. & Bialek, W. Weak pairwise correlations imply strongly correlated network states in a neural population. *Nature* **440**, 1007–1012 (2006).

[9] Buzsáki, G. & Mizuseki, K. The log-dynamic brain: how skewed distributions affect network operations. *Nat. Rev. Neurosci.* **15**, 264–278 (2014).

[10] Gal, E. *et al.* Rich cell-type-specific network topology in neocortical microcircuitry. *Nat. Neurosci.* **20**, 1004 (2017).

[11] Adamic, L. A. & Glance, N. The political blogosphere and the 2004 us election: divided they blog. In *Proceedings of the 3rd international workshop on Link discovery*, 36–43 (2005).

[12] Zou, Y., Pereira, T., Small, M., Liu, Z. & Kurths, J. Basin of attraction determines hysteresis in explosive synchronization. *Phys. Rev. Lett.* **112**, 114102 (2014).

[13] Rodrigues, F. A., Peron, T. K. D., Ji, P. & Kurths, J. The kuramoto model in complex networks. *Phys. Rep.* **610**, 1–98 (2016).

[14] Pereira, T., van Strien, S. & Tanzi, M. Heterogeneously coupled maps: hub dynamics and emergence across connectivity layers. *J. Eur. Math. Soc.* **22**, 2183–2252 (2020).

[15] Nishikawa, T., Motter, A. E., Lai, Y.-C. & Hoppensteadt, F. C. Heterogeneity in oscillator networks: Are smaller worlds easier to synchronize? *Phys. Rev. Lett.* **91**, 014101 (2003).

[16] Fries, P., Reynolds, J. H., Rorie, A. E. & Desimone, R. Modulation of oscillatory neuronal synchronization by selective visual attention. *Science* **291**, 1560–1563 (2001).

[17] Bruno, R. M. & Sakmann, B. Cortex is driven by weak but synchronously active thalamocortical synapses. *Science* **312**, 1622–1627 (2006).

[18] Morgan, R. J. & Soltesz, I. Nonrandom connectivity of the epileptic dentate gyrus predicts a major role for neuronal hubs in seizures. *Proc. Natl. Acad. Sci. USA* **105**, 6179–6184 (2008).

[19] Zamora-López, G., Zhou, C. & Kurths, J. Cortical hubs form a module for multisensory integration on top of the hierarchy of cortical networks. *Front. Neuroinf.* **4**, 1 (2010).

[20] Pikovsky, A. S. & Kurths, J. Coherence resonance in a noise-driven excitable system. *Phys. Rev. Lett.* **78**, 775 (1997).

[21] Semenova, N., Zakharova, A., Anishchenko, V. & Schöll, E. Coherence-resonance chimeras in a network of excitable elements. *Phys. Rev. Lett.* **117**, 014102 (2016).

[22] Ushakov, O. *et al.* Coherence resonance near a hopf bifurcation. *Phys. Rev. Lett.* **95**, 123903 (2005).

[23] Lindner, B., Garcıa-Ojalvo, J., Neiman, A. & Schimansky-Geier, L. Effects of noise in excitable systems. *Phys. Rep.* **392**, 321–424 (2004).

[24] Acebrón, J. A., Bonilla, L. L., Vicente, C. J. P., Ritort, F. & Spigler, R. The kuramoto model: A simple paradigm for synchronization phenomena. *Rev. Mod. Phys.* **77**, 137 (2005).

[25] Stankovski, T., Pereira, T., McClintock, P. V. & Stefanovska, A. Coupling functions: universal insights into dynamical interaction mechanisms. *Rev. Mod. Phys.* **89**, 045001 (2017).

[26] Montbrió, E. & Pazó, D. Shear diversity prevents collective synchronization. *Phys. Rev. Lett.* **106**, 254101 (2011).

[27] Ko, T.-W. & Ermentrout, G. B. Partially locked states in coupled oscillators due to inhomogeneous coupling. *Phys. Rev. E* **78**, 016203 (2008).

[28] Sakaguchi, H. & Kuramoto, Y. A soluble active rotater model showing phase transitions via mutual entertainment. *Progress of Theoretical Physics* **76**, 576–581 (1986).

[29] Omel'chenko, E. & Wolfrum, M. Nonuniversal transitions to synchrony in the sakaguchi-kuramoto model. *Phys. Rev. Lett.* **109**, 164101 (2012).

[30] Pimenova, A. V., Goldobin, D. S., Rosenblum, M. & Pikovsky, A. Interplay of coupling and common noise at the transition to synchrony in oscillator populations. *Sci. Rep.* **6**, 38518 (2016).

[31] Gammaitoni, L., Hänggi, P., Jung, P. & Marchesoni, F. Stochastic resonance. *Rev. Mod. Phys.* **70**, 223–287 (1998).

[32] Giusti, C., Ghrist, R. & Bassett, D. S. Twos company, three (or more) is a simplex. *J. Comp. Neurosci.* **41**, 1–14 (2016).

[33] Vlasov, V., Zou, Y. & Pereira, T. Explosive synchronization is discontinuous. *Phys. Rev. E* **92**, 012904 (2015).

[34] Ott, E. & Antonsen, T. M. Low dimensional behavior of large systems of globally coupled oscillators. *Chaos* **18**, 037113 (2008).

[35] Tyulkina, I. V., Goldobin, D. S., Klimenko, L. S. & Pikovsky, A. Dynamics of noisy oscillator populations beyond the ott-antonsen ansatz. *Phys. Rev. Lett.* **120**, 264101 (2018).

[36] Tönjes, R. & Pikovsky, A. Low-dimensional description for ensembles of identical phase oscillators subject to cauchy noise. *arXiv:2009.04725* (2020).

[37] Risken, H. *Fokker-Planck Equation* (Springer-Verlag, Berlin, 1989).

[38] Stratonovich, R. L. *Topics in the theory of random noise*, vol. 1 (Gordon and Breach, New York, 1963).

Supplementary Material

Coherence resonance in influencer networks

Ralf Tönjes[1], Carlos Fiore[2] and Tiago Pereira[3,4]

[1]*Institute for Physics and Astronomy, University of Potsdam, Karl-Liebknecht-Str. 24, 14476 Potsdam, Germany*

[2]*Instituto de Física, Universidade de São Paulo, São Paulo, Brazil*

[3]*Instituto de Ciências Matemáticas e Computação, Universidade de São Paulo, São Carlos, São Paulo, Brazil*

[4]*Department of Mathematics, Imperial College London, SW7 2AZ, London, UK*

Contents

A Example of transformation into canonical form **2**

B Hyper-graph **2**

C Impact of follower heterogeneity and noise **4**

D Examples of coherence resonance in influencer networks **5**

E Effective diffusion **5**

F Comparison of reduced models **7**

G Movies 8

A Example of transformation into canonical form

We start with weakly non-identical ($\omega_0 = 2.0$, $\Delta\omega/\omega_0 < 10\%$), weakly coupled ($\lambda_0 = 0.01$, $\beta = 10$) phase oscillators with moderately large shear ($c_0 = 0.5$) strong noise ($D = 1.4$) in the hubs and weak noise (or weak frequency heterogeneity) in the followers ($D_0 = 2 \times 10^{-4}$).

$$\begin{aligned}
\dot{\vartheta}_n &= 2.0 + \frac{0.01}{\mu_n} \sum_m W_{nm} \left[\sin(\vartheta_m - \vartheta_n - \alpha) + 0.5\right] + \sqrt{4 \times 10^{-4}} \xi_n \\
\dot{\vartheta}_{\text{hub}} &= 2.135 + \frac{0.1}{\mu_{\text{hub}}} \sum_m W_{\text{hub}\ m} \left[\sin(\vartheta_m - \vartheta_{\text{hub}} - \alpha) + 0.5\right] + \sqrt{2 \times 1.4} \xi_{\text{hub}}
\end{aligned}$$

After going into a co-rotating reference frame, where the follower mean natural frequency is zero $\vartheta \mapsto \vartheta - (\omega_0 + \lambda_0 c_0)t$ and a change of time scale $t \mapsto \lambda_0 t$, we obtain

$$\begin{aligned}
\dot{\vartheta}_n &= \frac{1}{\mu_n} \sum_m W_{nm} \sin(\vartheta_m - \vartheta_n - \alpha) + \sqrt{2 \times 0.02} \tilde{\zeta}_n \\
\dot{\vartheta}_{\text{hub}} &= 18 \left[1 + \frac{10}{18} \frac{1}{\mu_{\text{hub}}} \sum_m W_{\text{hub}\ m} \sin(\vartheta_m - \vartheta_{\text{hub}} - \alpha)\right] + \sqrt{2 \times \frac{140}{18} 18} \tilde{\zeta}_{\text{hub}}
\end{aligned}$$

The effective dimensionless parameters for this system are $\Delta\Omega/\lambda_0 = 18$, $\Lambda = 10/18$ and $q = 140/18 \approx 7$. The large noise strength $D/\lambda_0 = 140$ is only large in units of $\lambda_0 = 0.01$. Here, $D = 1.4$ is already much larger than the optimal noise strength $D_{\text{opt}} = 0.18$.

B Hyper-graph

We discuss the hyper-graph structure emerging at the macroscopic interaction of mean-fields. We will provide illustration of an influencer network with seven partitions of equal size connected through three influencers. After the

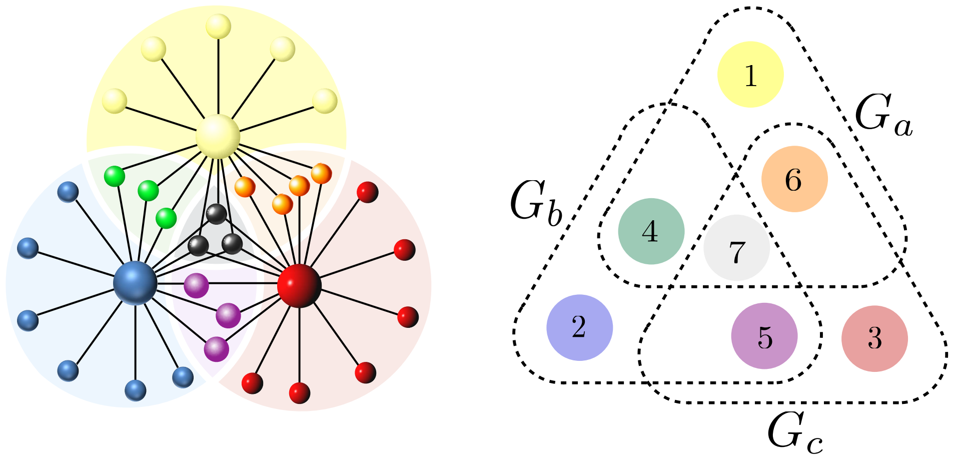

Figure B.1: **Three-influencer network and hyper-network**. Left panel, a network with three influencers. Right panel, a hyper-network of seven partitions connected by three hyper-edges.

Ott-Antonsen reduction of the partition mean-field dynamics and after averaging over the fast influencers, the resulting mean-field equations have the following form

$$
\begin{aligned}
\dot{Z}_1 = F\left(Z_1, G_a\right), \quad \dot{Z}_2 &= F\left(Z_2, G_b\right), \quad \dot{Z}_3 = F\left(Z_3, G_c\right), \\
\dot{Z}_4 = F\left(Z_4, \frac{1}{2}G_a + \frac{1}{2}G_b\right), \quad \dot{Z}_5 &= F\left(Z_5, \frac{1}{2}G_b + \frac{1}{2}G_c\right), \quad \dot{Z}_6 = F\left(Z_6, \frac{1}{2}G_c + \frac{1}{2}G_a\right), \\
\dot{Z}_7 &= F\left(Z_7, \frac{1}{3}G_a + \frac{1}{3}G_b + \frac{1}{3}G_c\right)
\end{aligned}
$$

with coupling functions

$$
\begin{aligned}
G_a &= G\left(\frac{1}{4}Z_1 + \frac{1}{4}Z_4 + \frac{1}{4}Z_6 + \frac{1}{4}Z_7; \Lambda_a, q_a\right) \\
G_b &= G\left(\frac{1}{4}Z_2 + \frac{1}{4}Z_4 + \frac{1}{4}Z_5 + \frac{1}{4}Z_7; \Lambda_b, q_b\right) \\
G_c &= G\left(\frac{1}{4}Z_3 + \frac{1}{4}Z_5 + \frac{1}{4}Z_6 + \frac{1}{4}Z_7; \Lambda_c, q_c\right)
\end{aligned}
$$

each connecting four partition mean-fields, and with Riccati dynamics

$$
F(Z, G) = \left(\frac{e^{-i\alpha}}{2}G - \frac{\gamma_0}{\lambda_0}Z - \frac{e^{i\alpha}}{2}\bar{G}Z^2\right). \tag{B.1}
$$

C Impact of follower heterogeneity and noise

In the main body of the manuscript we presented predictions and simulations with fixed values for frequency heterogeneity $\gamma_0/\lambda_0 = 0.02$ or noise strength $D_0/\lambda_0 = 0.02$ in the followers. The influence of these quantities on the coherence resonance can be seen in the mean-field analysis with slow-fast approximation. The frequency heterogeneity can be expressed as a function of all other system parameters, in particular the effective noise strength q and the global order parameter R. The contour plot of γ_0/λ_0 as a function of q and R shows the resonance curves for constant follower heterogeneity (see Fig. C.1).

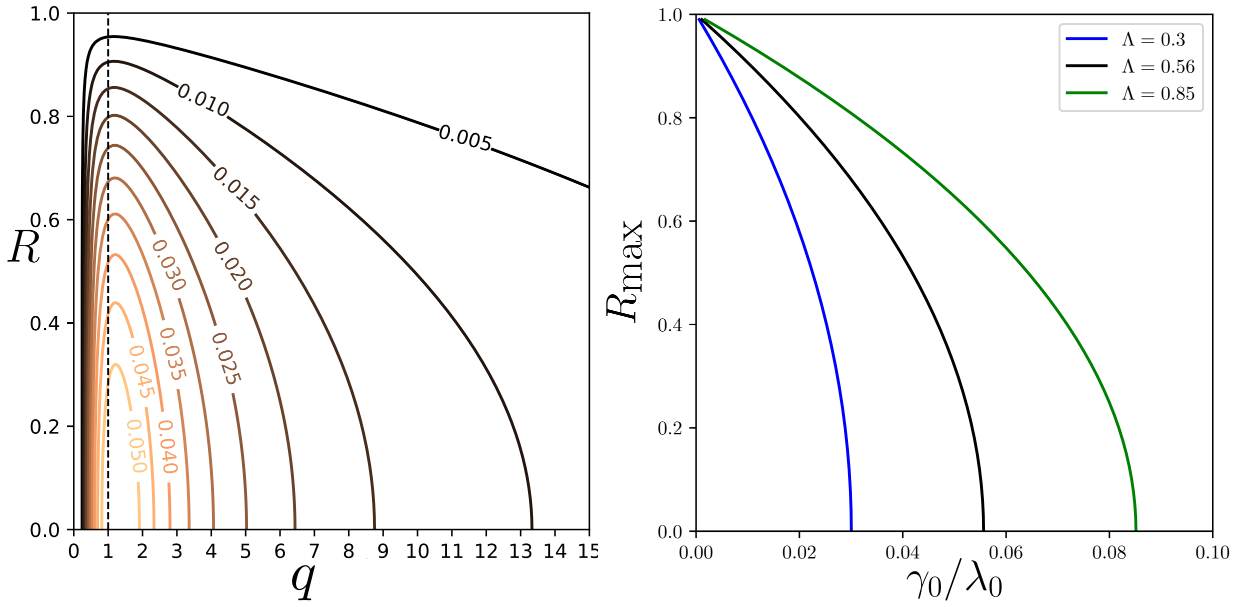

Figure C.1: **Effect of follower frequency heterogeneity on the coherence resonance**. The left panel show resonance curves of the order parameter as a function of effective noise q in the influencers, with effective coupling $\Lambda = 0.56$. Shown are the level sets of the right-handside. of Eq. (26), which correspond to different frequency heterogeneities γ_0/λ_0 in the followers, as indicated by the values in the labels. Using Gaussian white noise of equal strengths D_0/λ_0 instead of Lorentzian frequency distribution for follower frequencies of width γ_0/λ_0 results in similar resonance curves. In the right panel, we show the maximum value of the order parameter as a function of the frequency heterogeneity for different values of effective coupling strength Λ.

D Examples of coherence resonance in influencer networks

We show three examples of coherence resonance in Fig. D.1. For the three-influencer network motif described in Section B, we choose each partition with 100 nodes and undirected connections. We show an influencer network of 3000 nodes with 100 influencers. Every node (also the influencers) is connected symmetrically to $k+1$ randomly selected influencers where k is geometrically distributed with mean $10/6$. Since there are no direct links between followers, all connections have the weight $W_{mn} = 1$. For the directed hyperlink network of 1033 political weblogs after the 2004 US election, we choose the top 8 in-degree nodes as influencers. All nodes with zero out-degree have been removed. Phase coupling in the oscillator dynamics is realized in the opposite direction of the hyperlinks [1]. The time scale separation is $\Delta\Omega/\lambda_0 = 18$, $\beta = 10$ ($\Lambda = 10/18$). The noise strength in the followers is $D_0/\lambda_0 = 0.02$ in the three-influencer network and $D_0/\lambda_0 = 0.04$ in the election blog network.

E Effective diffusion

Here, we give an estimate of the effective noise strength for random forces $c(t) = \lambda_0 \cos(\psi - \alpha)$ and $s(t) = \lambda_0 \sin(\psi - \alpha)$ when ψ is a drift-diffusion process

$$\psi(t+\tau) = \psi(t) + \Omega\tau + W_D(\tau) \pmod{2\pi} \tag{E.1}$$

on the circle with constant velocity Ω, Brownian diffusion $W_D(\tau)$, and diffusion constant D. Such forces act on the follower phases coupled to influencers with phase ψ when the global order parameter is zero, that is, in an incoherent state. The conditional probability density $p(\psi, t+\tau|\psi_0, t)$ is a wrapped normal distribution $p_{WN}(\psi; \mu, \sigma^2)$ with mean $\mu = \psi_0 + \Omega\tau$ and variance $\sigma^2 = 2D\tau$, which in the limit $\tau \to \infty$ becomes a stationary, uniform distribution on the circle. The complex autocorrelation function

$$C(\tau) = \lambda_0^2 \left\langle e^{-i\psi(t)} \cdot e^{i\psi(t+\tau)} \right\rangle_t \tag{E.2}$$

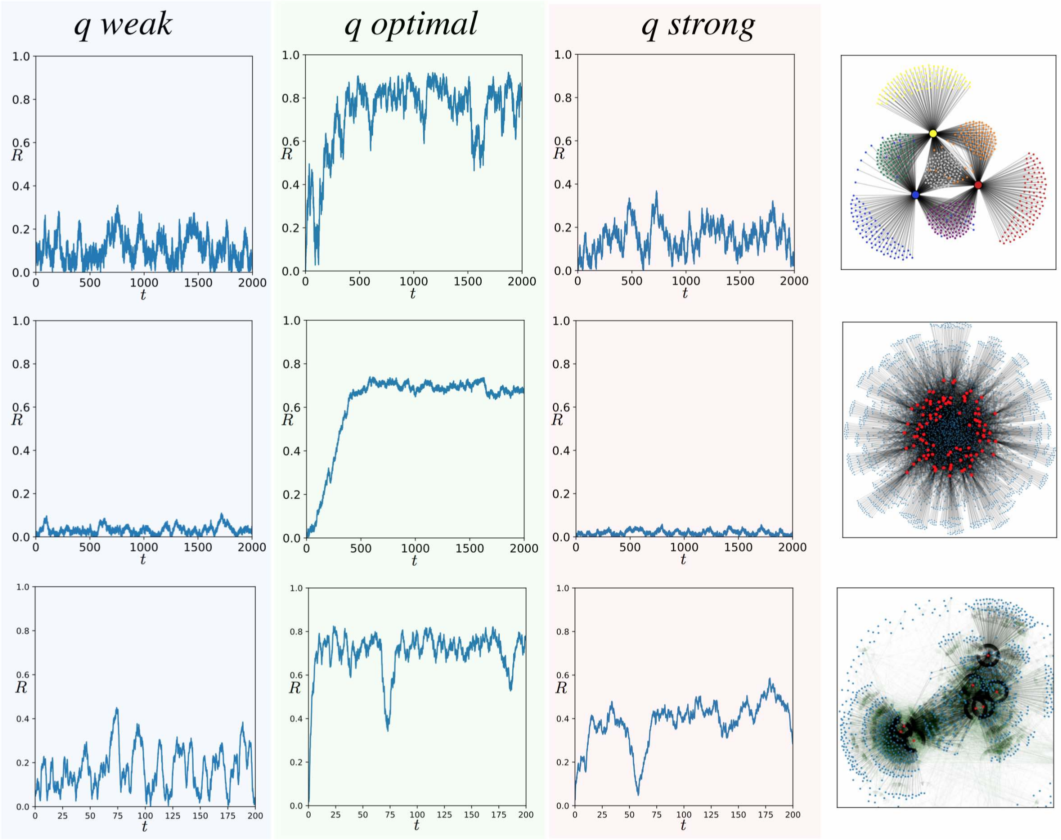

Figure D.1: **Coherence resonance of the order parameter for different influencer networks**. We show the time series of the order parameter R for three values of noise intensity in the influencers and the network in the corresponding row. Parameters are $\Delta\Omega/\lambda_0 = 10/18$ and $\Lambda = 10/18$. In the first row, we show the influencer network with three influencers and seven partitions as shown in Section B, with $D_0/\lambda_0 = 0.02$. In the second row, we show an influencer network with 3000 nodes including 100 influencers. In the third row, we show the directed hyperlink network of 1033 political weblogs after the 2004 US election [1] and the top 8-out degree nodes as influencers. Arrows indicate coupling direction which is in the opposite direction of the hyperlinks. The noise strength in the followers is $D_0/\lambda_0 = 0.04$. The noise intensity in the influencers for the simulations in the first three panel columns is weak $q = 0.1$, optimal $q = 1.0$, and strong $q = 10$.

is the sum of the autocorrelation functions of the two forces $c(t) = \lambda_0 \cos(\psi - \alpha)$ and $s(t) = \lambda_0 \sin(\psi - \alpha)$ in the real part and the cross-correlation function in the imaginary part. Replacing the time average by the average with respect to the conditional probability density and the average over the initial conditions ψ_0 with respect to the stationary probability density, the complex autocorrelation function has the form of the first circular moment of the wrapped

normal distribution

$$C(\tau) = \lambda_0^2 \left\langle e^{i(\Omega\tau + W_D(\tau))} \right\rangle_{p_{WN}} = \lambda_0^2 e^{(i\Omega - D)\tau}. \tag{E.3}$$

Because of the uniform stationary distribution, the autocorrelation functions of $c(t)$ and $s(t)$ are identical and both forces are uncorrelated; that is $\langle c(t)s(t)\rangle_t = 0$. The time integrals of the random forces are random variables that, according to the central limit theorem, have a variance that grows asymptotically linearly in time. The effective diffusion constant, which is half of the asymptotic speed of this growth, is the integral of the autocorrelation functions

$$D_{\text{eff}} = \frac{\lambda_0^2}{2} \int_0^\infty \text{Re}\left[e^{(i\Omega - D)\tau}\right] d\tau = \frac{\lambda_0^2}{2} \frac{D}{D^2 + \Omega^2} \tag{E.4}$$

Measuring time in units of $1/\lambda_0$, we obtain the expression in the Methods section of the manuscript.

F Comparison of reduced models

The Ott-Antonsen ansatz for ensembles of phase oscillators with Lorenzian frequency distribution and under common forcing in the zeroth and in the first harmonics results is an exact expression for the dynamics of the mean-field. The forcing can be stochastic if interpreted as Stratonovich stochastic differential equation. In the thermodynamic limit $N \to \infty$ but for finite dynamical frequency gap $\Delta\Omega/\lambda_0$, the fluctuations in the influencer phases lead to fluctuations in the partition mean-fields that are comparable to the partition mean-field fluctuations in the finite size network dynamics. For a large dynamical frequency gap, the effective noise strength of the stochastic forcing vanishes. In the slow-fast approximation, the followers are only subject to the average forces from the influencers, which in turn depend adiabatically on the partition mean-fields. While fluctuations are completely absent in the averaged dynamics, the average forces depend continuously on the influencer effective noise strength $q = D/\Delta\Omega$. Indeed, while for $\Delta\Omega/\lambda_0 = 18$ the averaged dynamics describes the equilibrium order parameters near the optimal noise strength reasonably well, for much larger time scale separation $\Delta\Omega/\lambda_0 = 198$ even the transient to equilibrium is reproduced robustly Fig. E.1.

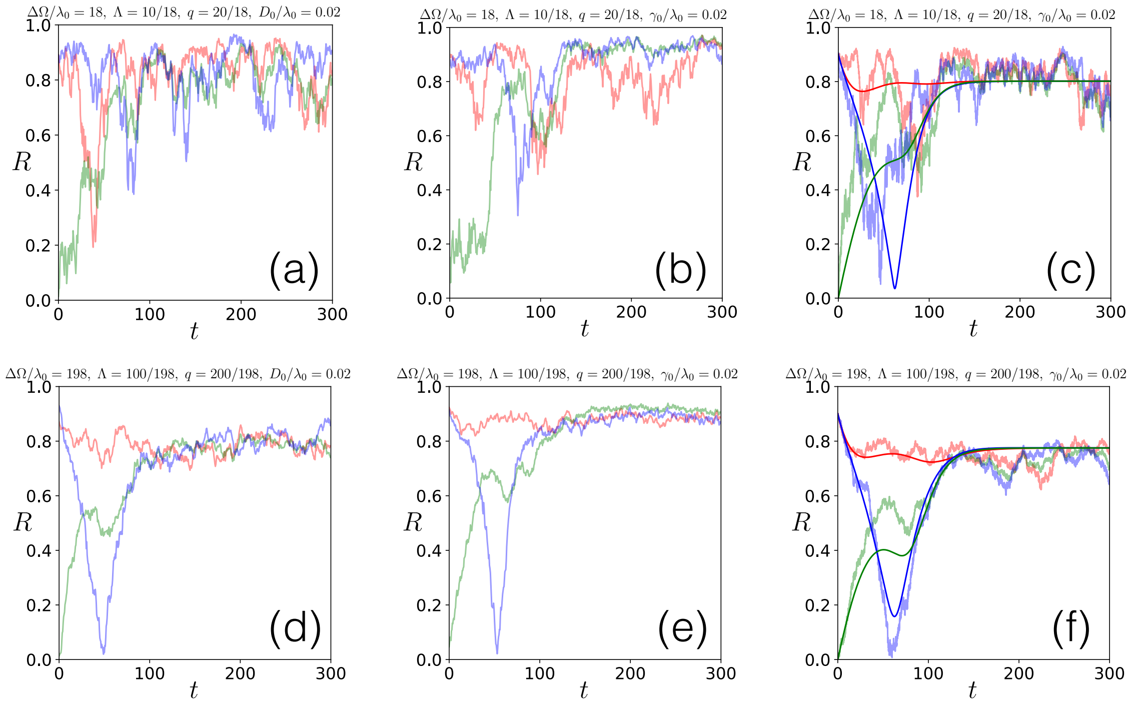

Figure E.1: **Time series of the order parameters** R_σ (R_1 red, R_2 green, R_3 blue) for the network in Figure 1 of the main manuscript with two influencers for (a)-(c) moderate time scale separation $\Delta\Omega/\lambda_0 = 18$, noise $q = 20/18$ and reduced coupling strength $\Lambda = 10/18$ and (d)-(f) large time scale separation $\Delta\Omega/\lambda_0 = 198$ with noise $q = 200/198$ and $\Lambda = 100/198$. Full network simulations with noise $D_0/\lambda_0 = 0.02$ in the followers are shown in (a) and (d). Full network simulations with frequency heterogeneity $\gamma_0/\lambda_0 = 0.02$ in the followers are shown in (b) and (e). In (c) and (f) we show simulations of the Ott-Antonsen mean-field equations under stochastic forcing by the influencers and of the averaged dynamics (smooth curves) obtained under the assumption of infinite time scale separation. The initial conditions are $Z_1 = 0.9\exp(i\pi/2)$, $Z_3 = 0.9\exp(-i\pi/4)$, $Z_3 = 0$, $\psi_a = \pi$ and $\psi_b = -\pi/2$. The drop in the order parameter R_3 is predicted by the averaged dynamics and can be observed in the full network simulations with noise D_0 or frequency heterogeneity γ_0.

G Movies

We simulate the influencer network as in Figure 1 of the main manuscript and generated two short movies. Parameters when not specified are as in Figure 1 (left panel). We show the phases on the vertical axis and the index of oscillators on the horizontal axis. The time evolution of the system reveals the formation of clusters and the time evolution of the

macroscopic fields. All relevant parameters are displayed in the movie. The movies are:

TwoInfluencer-gamma.mp4 display the dynamics of noiseless followers with frequency heterogeneity.

TwoInfluencer-Noisy.mp4 display the dynamics identical followers with subjected to independent noise.

References

[1] Adamic, L. A. & Glance, N. The political blogosphere and the 2004 us election: divided they blog. In *Proceedings of the 3rd international workshop on Link discovery*, 36–43 (2005).

Reviewer #3 (Remarks to the Author):

Recommendation: Accept With No Changes

Comments:
The authors addressed the main concerns from the reviews, the revised version of the manuscript appears to be good. In particular, the model and the parameters are now well-defined and presented in a clearer way. It looks ready for publication as far as I can tell.

Reply to REVIEWERS' COMMENTS

Reviewer #1 (Remarks to the Author):

The authors follow my suggestion and explain the phenomenon of coherence resonance right at the beginning of the manuscript. However, a few points should be improved:

- In [21] nonlocal coupling on a ring is considered, not all-to-all coupling (line 49)

    We thank the reviewer. We amended it and included Ref. [23] about all-to-all coupling. A description of previous work on coherence resonance was moved to the beginning of the introduction upon editorial request.

- Please specify the bifurcation type in the sentence: "...near a Hopf bifurcation [22]" (line 50)

    They investigate both supercritical and subcritical Hopf bifurcation

- Network dependent features (line 51-52) of coherence resonance have been previously investigated. For example, there is a resonance value of the interaction strength that is optimal for coherence resonance (see Ref. [1] below). Several other works have also shown that coherence resonance can be enhanced by choosing appropriate coupling strengths. For example, in Refs. [2] and [3], it was shown that some choices of coupling strength increase coherence resonance in an array of non-identical FitzHugh-Nagumo oscillators; in Ref. [4], a similar feature was observed in the case of weighted scale-free networks.

    Thank you for the remarks we have amended the text and included the first reference suggested by the referee as another example for coherence resonance in networks of excitable units.

With these minor amendments, I recommend the manuscript for publication.

    We thank the reviewer for the recommendation.
